# Cellulose binding and the timing of expression influence protein targeting to the double-layered cyst wall of *Acanthamoeba*

**DOI:** 10.1128/msphere.00466-24

**Published:** 2024-08-13

**Authors:** Bharath Kanakapura Sundararaj, Manish Goyal, John Samuelson

**Affiliations:** 1 Department of Molecular and Cell Biology, Boston University Goldman School of Dental Medicine, Boston, Massachusetts, USA; University at Buffalo, Buffalo, New York, USA

**Keywords:** *Acanthamoeba*, AlphaFold structure, cellulose binding, cyst wall proteins, domain evolution, promoter swap

## Abstract

The cyst wall of the eye pathogen *Acanthamoeba castellanii* contains cellulose and has ectocyst and endocyst layers connected by conical ostioles. Cyst walls contain families of lectins that localize to the ectocyst layer (Jonah) or the endocyst layer and ostioles (Luke and Leo). How lectins and an abundant laccase bind cellulose and why proteins go to locations in the wall are not known and are the focus of the studies here. Structural predictions identified β-jelly-roll folds (BJRFs) of Luke and sets of four disulfide knots (4DKs) of Leo, each of which contains linear arrays of aromatic amino acids, also present in carbohydrate-binding modules of bacterial and plant endocellulases. Ala mutations showed that these aromatics are necessary for cellulose binding and proper localization of Luke and Leo in the *Acanthamoeba* cyst wall. BJRFs of Luke, 4DKs of Leo, a single β-helical fold (BHF) of Jonah, and a copper oxidase domain of the laccase each bind to glycopolymers in both layers of deproteinated cyst walls. Promoter swaps showed that ectocyst localization does not just correlate with but is caused by early encystation-specific expression, while localization in the endocyst layer and ostioles is caused by later expression. Evolutionary studies showed distinct modes of assembly of duplicated domains in Luke, Leo, and Jonah lectins and suggested Jonah BHFs originated from bacteria, Luke BJRFs share common ancestry with slime molds, while 4DKs of Leo are unique to *Acanthamoeba*.

**IMPORTANCE:**

*Acanthamoebae* is the only human parasite with cellulose in its cyst wall and conical ostioles that connect its inner and outer layers. Cyst walls are important virulence factors because they make *Acanthamoebae* resistant to surface disinfectants, hand sanitizers, contact lens sterilizers, and antibiotics applied to the eye. The goal here was to understand better how proteins are targeted to specific locations in the cyst wall. To this end, we identified three new proteins in the outer layer of the cyst wall, which may be targets for diagnostic antibodies in corneal scrapings. We used structural predictions and mutated proteins to show linear arrays of aromatic amino acids of two unrelated wall proteins are necessary for binding cellulose and proper wall localization. We showed early expression during encystation causes proteins to localize to the outer layer, while later expression causes proteins to localize to the inner layer and the ostioles.

## INTRODUCTION

*Acanthamoeba* keratitis (AK), which leads to scarring and blindness, if not successfully treated, is caused by free-living amoebae, named for acanthopods (spikes) on the surface of trophozoites (1–3). In immunocompromised patients, *Acanthamoebae* may cause encephalitis, which is frequently fatal (4). AK is associated with corneal trauma in the Middle East, South Asia, and Africa, where water for handwashing is often scarce (5–9). AK is associated with contact lens use in the United States, Europe, and Australia, and so *Acanthamoeba* is on the NIAID list of Emerging Infectious Diseases/Pathogens (3, 10–12). Climate warming increases the growth of *Acanthamoebae* in untreated water, causing a greater risk of human exposure and disease (13).

Cyst walls, which form when trophozoites are starved of nutrients, protect free-living *Acanthamoebae* from osmotic shock in freshwater or drying in the air (14, 15). The cyst wall also makes *Acanthamoebae* resistant to disinfectants used to clean surfaces, alcohol-based hand sanitizers, sterilizing agents in contact lens solutions, and/or antibiotics applied to the eye (16–20). Nearly 50 years ago, cellulose (β-1,4- linked glucose) was identified in *Acanthamoeba* cyst walls, which have an ectocyst layer (outer) and an endocyst layer (inner) connected by conical ostioles (21, 22). The approximately nine ostioles per cyst wall have a narrow outer layer and numerous splits in the inner layer. During excystation, the ostioles break down, allowing the trophozoite to escape and the life cycle to continue. Because *Acanthamoeba* has a chitin synthase and wheat germ agglutinin (WGA) binds to cyst walls, it is likely that chitin (β-1,4-linked GlcNAc) is also present (23, 24).

No cyst wall proteins were known until we enriched with density gradients cyst walls from the Neff strain of *Acanthamoeba castellanii* (Ac) and identified using mass spectrometry three large families of lectins, which were named Jonah, Luke, and Leo (25–27). When a representative protein from each family was tagged with green fluorescent protein (GFP) and expressed under its own promoter in transfected Ac, Jonah-1 localized was made early in encystation and localized to the ectocyst layer, while Luke-2 and Leo-A were made later in encystation and localized to the endocyst layer and ostioles (28, 29).

Here, we performed four sets of studies to understand better the structure, localization, and origin of Jonah, Luke, and Leo lectins, as well as a laccase that was abundant in Ac wall preparations (27). First, we used AlphaFold and Foldseek to predict the structures of Luke and Leo lectins (30–32) and made alanine mutations to test whether linear arrays of aromatic amino acids bind cellulose and/or are necessary for proper localization of lectins in the cyst wall (33–38). Second, we determined the localization of additional Leo and Jonah lectins, which differ in structure from those previously tested, as well as the laccase. Third, we used double labels, deproteinated walls, and promoter swaps to dissect why proteins localize to the ectocyst layer versus the endocyst layer and ostioles. Fourth, we used phylogenetic trees to suggest how wall proteins with one, two, or three similar domains were assembled and duplicated.

## RESULTS

### Luke lectins are composed of two or three β-jelly-roll folds, each of which contains linear arrays of three aromatic amino acids that bind cellulose and target the protein to the endocyst layer and ostioles

**Fig 1:**
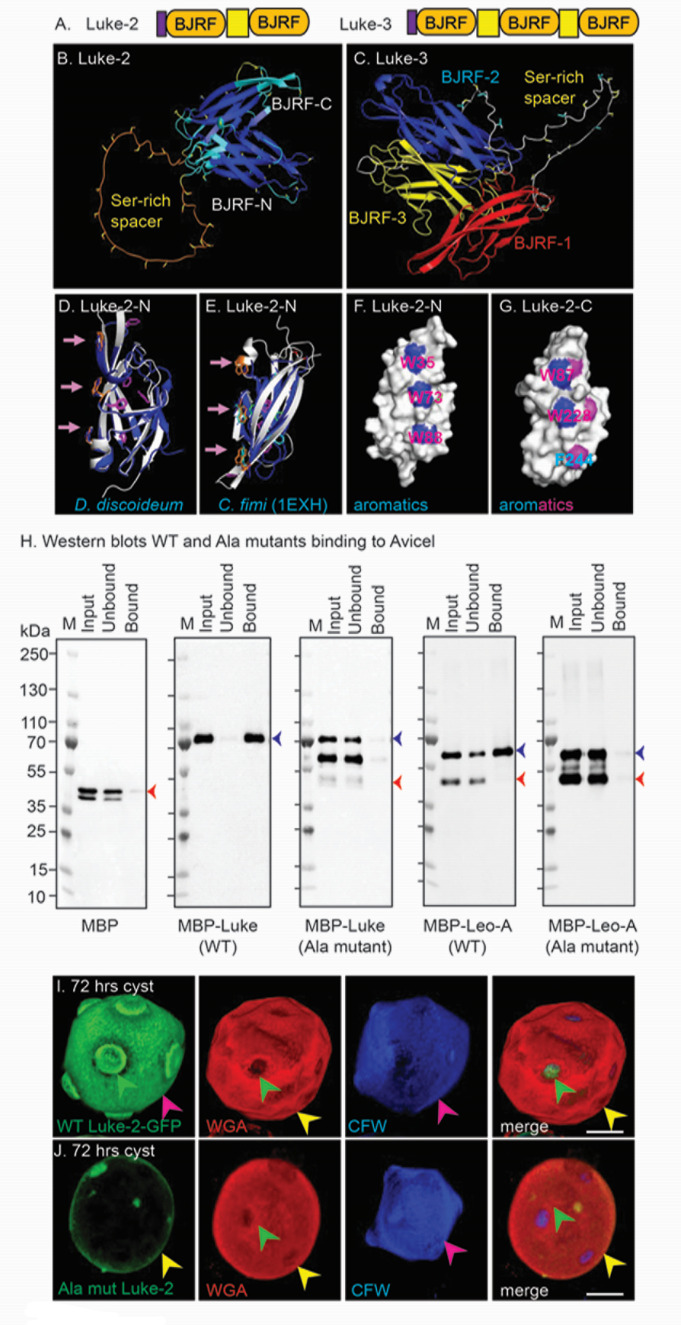
A linear array of three aromatic amino acids in the β-jelly-roll folds (BJRFs) of Luke-2 bind cellulose and direct the protein to the endocyst layer and ostioles. (**A**) Drawings show Luke-2 and Luke-3 contain a signal peptide (purple) and two or three BJRFs (orange), respectively, which are separated by Ser-rich spacers (yellow). (**B**) AlphaFold structure with the confidence colored shows that Luke-2 contains two BJRFs, which are separated by a 47-aa, unstructured spacer rich in Ser (yellow). (**C**) Luke-3 has three BJRFs separated by 42- and 33-aa long, unstructured spacers that are also Ser-rich. (**D**) Foldseek shows the N-half BJRF of Luke-2 shares a similar structure with a BJRF of *Dictyostelium* (DDB_G0292224) wall protein (*E*-value = 4.99e−7 for foldseek, RMSD = 1.79, and 30.3% identity over a 121-amino acid overlap), which together suggest a relatively recent common ancestry. Importantly, the BJRFs of Luke-2 and the *Dictyostelium* wall protein each have three aromatic amino acids that form a linear array (pink arrows). (**E**) The N-half BJRF of Luke-2 also shares three aromatic acids with the CBM2 of a *Cellulomonas fimi* endoglucanase (blue), the structure of which has been solved (PDB 1EXH). The *E*-value for the N-terminal BJRF of Luke-2 and the CBM2 of *C. fimi* is 9.72e−5; the RMSD is 5.01; and the positional is 16% identity over a 105-aa overlap, which together suggest a more remote ancestry. (**F and G**) Surface views of N- and C-half BJRFs of Luke-2 highlight linear arrays of Trp (blue) and Phe (pink), which were mutated to Ala in MBP fusions made in the periplasm of *E. coli* or in GFP-tagged proteins expressed in transfected Ac that are encysting. (**H**) Western blots show that MBP alone (red arrow) fails to bind to Avicel cellulose, while full-length MBP–Luke-2 and MBP–Leo-A fusion proteins (blue arrows) each bind well to Avicel cellulose. In contrast, cellulose binding is lost when six aromatics are mutated to Ala in both Luke-2 and Leo-A. The extra lower molecular weight bands in the WT and Ala mutants of Leo-A are likely MBP only (red arrows), as only the higher molecular weight intact WT fusion protein binds to Avicel. (**I**) Confocal microscopy shows WT Luke-2–GFP localizes to the endocyst layer, which is labeled with CFW (pink arrowheads), and forms a flat ring around ostioles (green arrowheads). Here and in Fig. 2 to 4, the ectocyst layer (yellow arrowheads) is labeled with WGA that binds to chitin. (**J**) When five Tyr and a Phe are mutated to Ala, Ala-mut Luke-2–GFP no longer localizes to endocyst layer or ostioles. These experiments support the idea that the Ala-mutant of Luke-2 mislocalizes in cyst walls because of the loss of cellulose binding. Confocal micrographs here and in Fig. 2 to 5 were shot with a ×60 objective, and 3D reconstructions were made from 0.1-µm optical sections. Scale bars for panels I and J are each 5 µm.

Structure predictions showed Luke-2 (ACA1_377670) and Luke-3 (ACA1_245650) have two and three β-jelly-roll folds (BJRFs), respectively, each of which has a disulfide bond linking its beginning and end (Fig. 1A through C) (25–27, 30). The BJRFs of Luke lectins are separated by short unstructured domains enriched in Ser. The BJRFs of Luke-2 and a predicted *Dictyostelium* cellulose-binding protein (DDB_G0292224) each contain a linear array of three aromatic amino acids also present in CBM2 of an endocellulase of a soil bacterium (*Cellulomonas fimi*), the structure of which has been solved (PDB 1EXH) (Fig. 1D and E) (39, 40). Ala mutations of these three aromatic amino acids in CBM2 and in CBM49 of a plant endocellulase, which is closely related, show they are the binding sites for cellulose (33–38). Here we mutated to Ala two sets of three aromatics (W35, W73, and W88 and W187, W228, and F244) of Luke-2 (Fig. 1F and G), which were fused to maltose-binding protein (MBP) and made in the periplasm of *Escherichia coli* (27, 41, 42). Western blots, which were repeated three times, showed wild-type Luke-2 binds well to Avicel cellulose, while MBP alone and Luke-2 plus Ala mutations fail to bind to cellulose (Fig. 1H). Here, we assume, but have not proven, that the Ala mutations of Luke-2 and Leo-A are properly folded. Luke-2-GFP plus Ala mutations no longer localize to the endocyst layer and ostioles, which were labeled with calcofluor white (CFW) that binds to cellulose (Fig. 1I and J) (27, 43). N.B., the relative intensity of Luke-2-GFP in the endocyst layer and ostioles, may vary from cell to cell (see also Fig. 3J). Further, there appeared to be no dominant-negative effect of Ala mutants of Luke-2-GFP or Leo-A-GFP on cell wall formation. Quantitation of three experimental repeats of this experiment is shown in Fig. 5O. Here, and in many, but not all confocal micrographs, the ectocyst layer is marked by WGA, which binds chitin (23, 27, 44). These results suggest that the localization of Luke-2 in the endocyst layer and ostioles is dependent upon its binding to cellulose, while binding to chitin is also possible.

**Fig 2:**
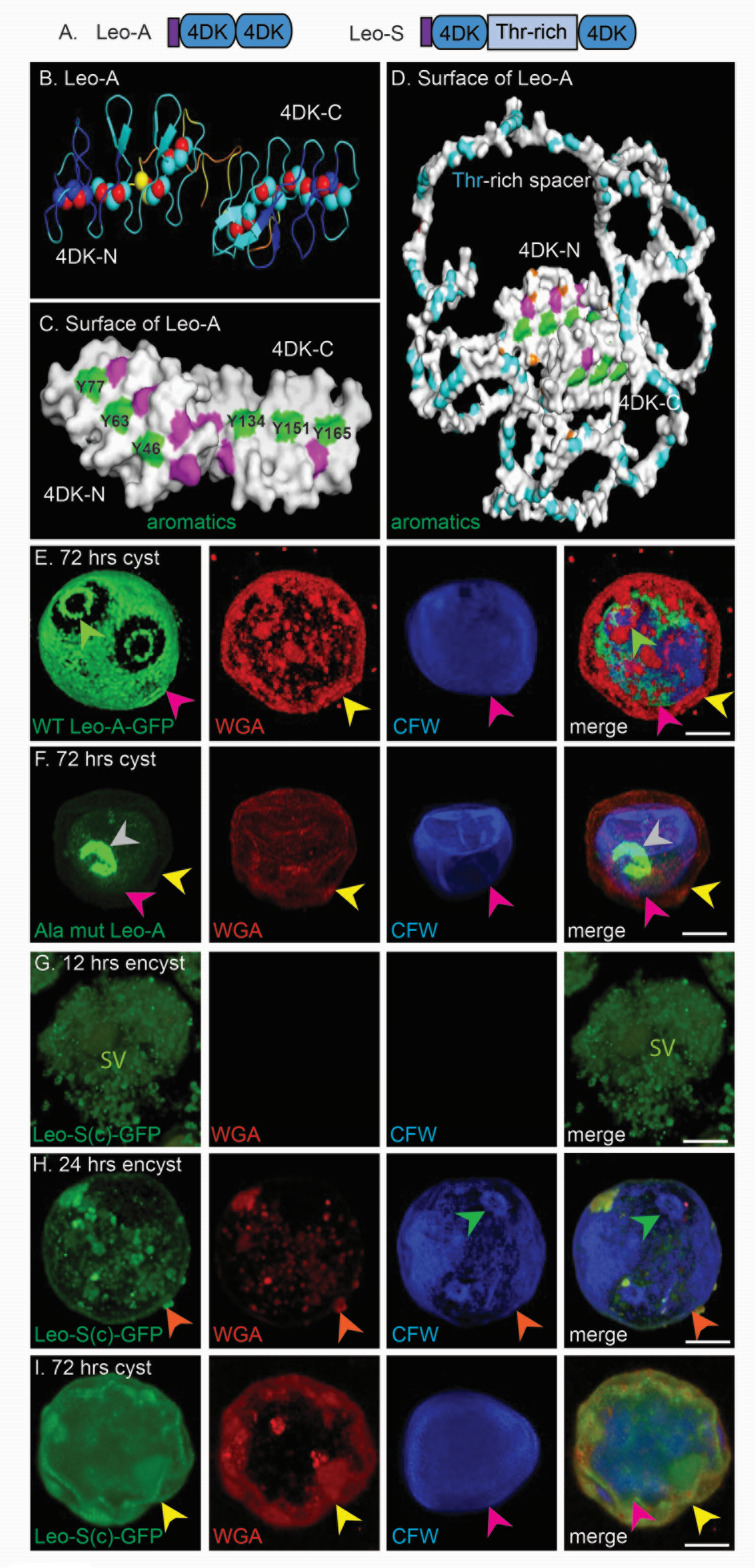
Although unique sets of four disulfide knots (4DKs) of Leo lectins show no resemblance to BJRFs of Luke, linear arrays of three aromatic amino acids bind cellulose and direct the protein to the endocyst layer and ostioles (Leo-A) or ectocyst layer [Leo-S(c)]. (**A**) Drawings show Leo-A contains a signal peptide (purple) and two adjacent sets of four 4DKs (blue), while Leo-S(c), which was corrected by the new transcriptome, contains a Thr-rich spacer (light blue) between sets of 4DKs. (**B**) AlphaFold with the confidence colored shows Leo-A is composed of two adjacent sets of 4DKs (red spheres), which connect short, parallel loops. Foldseek had no hit with 4DKs of Leo, suggesting that the domain is unique to Ac or is present only in organisms not part of Foldseek searches. (**C**) Surface view of Leo-A shows three Tyr residues (green) form a linear array in each 4DK, which were mutated to Ala to test the effects on binding to Avicel cellulose by MBP-Leo-A (see Fig. 1H). (**D**) Surface view of Leo-S(c) shows a 252-aa-long unstructured, spacer rich in Thr (blue) between 4DKs, each of which contains linearly arrayed Tyr residues. (**E**) Confocal microscopy shows WT Leo-A localizes to the endocyst layer (pink arrowheads) and forms a flat ring around ostioles (green arrowheads). (**F**) When six Tyr are mutated to Ala, Ala-mut Leo-A (gray arrowheads) no longer localizes to the endocyst layer. The results here and in Fig. 1J support the idea that the Ala-mutant of Leo-A, like the Ala-mutant of Luke-2 (Fig. 1), mislocalizes in cyst walls because of the loss of cellulose binding. (**G**) Early in encystation (12 h), secretory vesicles (SV) containing Leo-S(c) fill the cytosol of Ac, which lack a cyst wall as shown by failure to label with WGA or CFW. (**H**) Later in encystation (24 h), Leo-S(c) has a somewhat patchy distribution in the single-layered wall, which now labels with WGA and CFW. (**I**) In mature cyst walls (72 h), Leo-S(c) has a homogeneous distribution in the ectocyst layer (yellow arrowheads).These results show that despite their shared 4DKs, Leo-S(c) and Leo-A do not localize to the same place in the cyst wall. Scale bars for panels E to I are each 5 µm.

**Fig 3:**
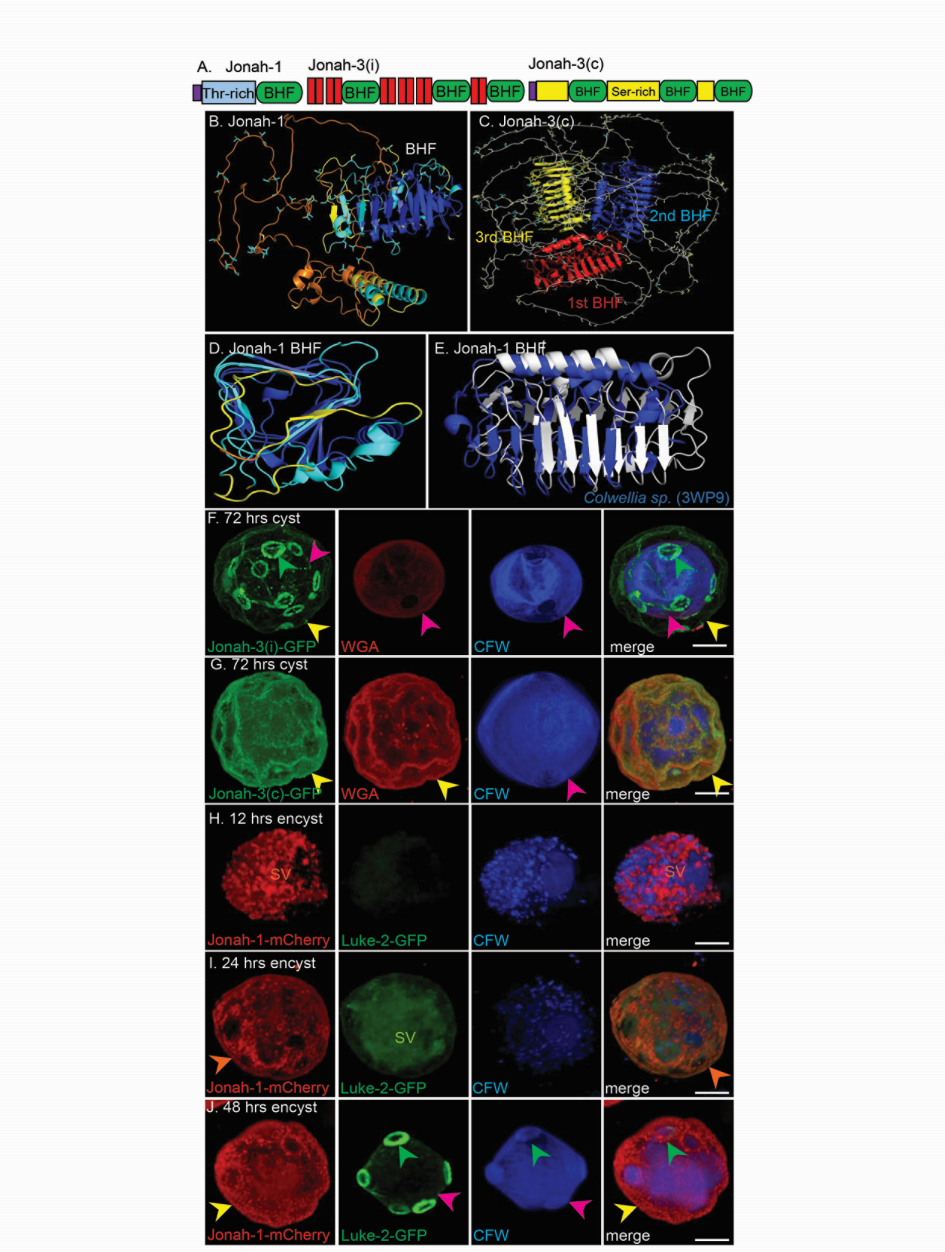
Jonah lectins, which have either one or three three-sided, β-helical folds (BHFs), like those of ice-binding proteins of Antarctic bacteria, are made early in encystation and localize to the ectocyst layer. (**A**) Drawings show that Jonah-1 contains a signal peptide (purple), followed by a Thr-rich spacer (light blue), and a single BHF (green). Jonah-3(i), which was incorrectly predicted by AmoebaDB, has 12 TMHs (red) and three BHFs, while Jonah-3(c), corrected by the new transcriptome, has three BHFs, three Ser-rich domains (yellow), and no TMHs. (**B**) AlphaFold structure with confidence colored shows that the Jonah-1 lectin is composed of a single three-sided, β-helical fold (BHF), as well as two α-helices (function unknown), and a 170-aa, unstructured domain rich in Thr (light blue). (**C**) Jonah-3-(**C**) has three BHFs (red, blue, and yellow) and 132-, 267-, and 308-aa long, unstructured domains rich in Ser (yellow). (**D**) End-view with confidence colored shows that the Jonah-1 BHF is three-sided (BHFs may also be two-sided). (**E**) Foldseek shows the Jonah-1 BHF (white) resembles the BHF of *Colwellia* sp. (blue), the structure of which has been solved (PDB 3WP9). The *E*-value is 5.10e−4; the RMSD is 9.21; and the sequence identity is 12% over a 245-aa overlap, which together suggest a distant shared ancestry. While surface views reveal numerous aromatic acids, none were in linear arrays so that we declined to make Ala mutations to test cellulose-binding sites. (**F**) Confocal microscopy shows Jonah-3(i), which was incorrectly predicted by AmoebaDB to contain 12 TMHs, forms rings around ostioles (green arrowheads) when expressed under its own promoter with a GFP tag. Here, the endocyst layer is marked by CFW and WGA (pink arrowheads), while the ectocyst layer (yellow arrowheads) is weakly labeled. (**G**) In contrast, Jonah-3(c)-GFP, which contains no TMHs, localizes to the ectocyst layer (yellow arrowheads). (**H**) After 12 h of encystation, Ac are filled with secretory vesicles (SV) containing Jonah-1–mCherry, as well as CFW-labeled vesicles most likely containing cellulose, while Luke-2-GFP is absent. (**I**) After 24 h of encystation, Jonah-1–mCherry localizes to the ectocyst layer (yellow arrowheads), while CFW and Luke-2-GFP are predominantly in secretory vesicles. (**J**) After 48 h of encystation, the cyst wall has the appearance of mature cysts with Jonah-1–mCherry in the ectocyst layer, Luke-2-GFP in ostioles and to a lesser extent in the endocyst layer, and CFW predominantly in the endocyst layer. These double labels, which clearly show that Jonah-1 is made early in encystation, while Luke-2 is made later, justify Jonah-1 and Luke-2 in the first promoter swap in Fig. 5. Scale bars for panels F to J are each 5 µm.

**Fig 4:**
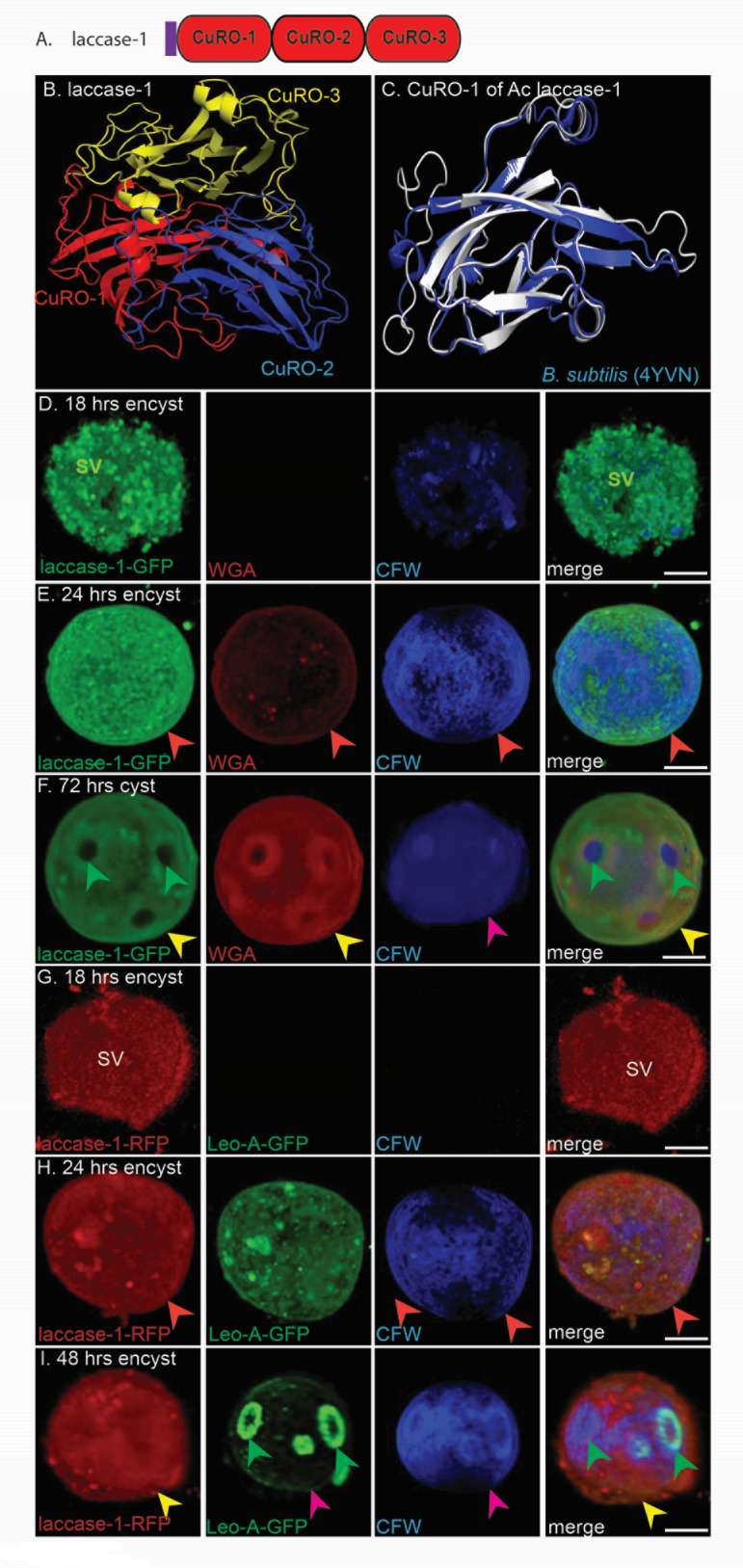
An abundant laccase, which resembles a bacterial spore coat protein, is made early in encystation and localizes to the ectocyst layer. (**A**) The Ac laccase-1 has signal peptide (purple) and three copper oxidase domains, separated by short, unstructured spacers lacking either Ser or Thr. (**B**) AlphaFold shows CuRO-1 (blue), CuRO-2 (red), and CuRO-3 (yellow) domains of Ac laccase-1, all of which are predicted with confidence (not shown). (**C**) Foldseek shows that CuRo-1 of the Ac laccase-1 closely resembles the first copper oxidase domain of *Bacillus subtilis* spore coat protein, which has been crystallized (PDB 4YVN). Typical for an enzyme, the *E*-value is 1.32e−59 and the RMSD = 2.75 with a sequence identity of 39% over a 505-aa overlap for three copper oxidase domains. (**D**) Confocal microscopy shows that after 18 h of encystation, secretory vesicles (SV) containing laccase-1-GFP fill the cytosol of Ac, which lacks a cyst wall as shown by the failure to label with WGA or CFW. (**E**) After 24 h of encystation, laccase-1-GFP has a homogeneous distribution in the single-layered wall, which labels with WGA and CFW. (**F**) In mature cyst walls (72 h), laccase-1-GFP has a homogeneous distribution in the ectocyst layer (yellow arrowheads) with ostioles appearing as dimples (green arrowheads). Double labels were used to compare the localization of laccase-1-RFP and Leo-A-GFP during encystation. (**G**) After 18 h of encystation, Ac are filled with secretory vesicles (SV) containing laccase-1-RFP, while Leo-A-GFP is absent. (**H**) After 24 h of encystation, laccase-1-RFP localizes to the ectocyst layer (yellow arrowheads), while CFW and Luke-2-GFP are predominantly in secretory vesicles. (**I**) After 48− h of encystation, the cyst wall has the appearance of mature cysts with laccase-1-RFP in the ectocyst layer, Leo-A-GFP in ostioles (green arrowheads) and to a lesser extent in the endocyst layer (pink arrowheads), and CFW predominantly in the endocyst layer. The clear demonstration here that laccase-1 is made early in encystation, while Leo-A is made later, justifies the use of laccase-1 and Leo-A in the second promoter swap in Fig. 5. Scale bars for panels D to I are each 5 µm.

**Fig 5:**
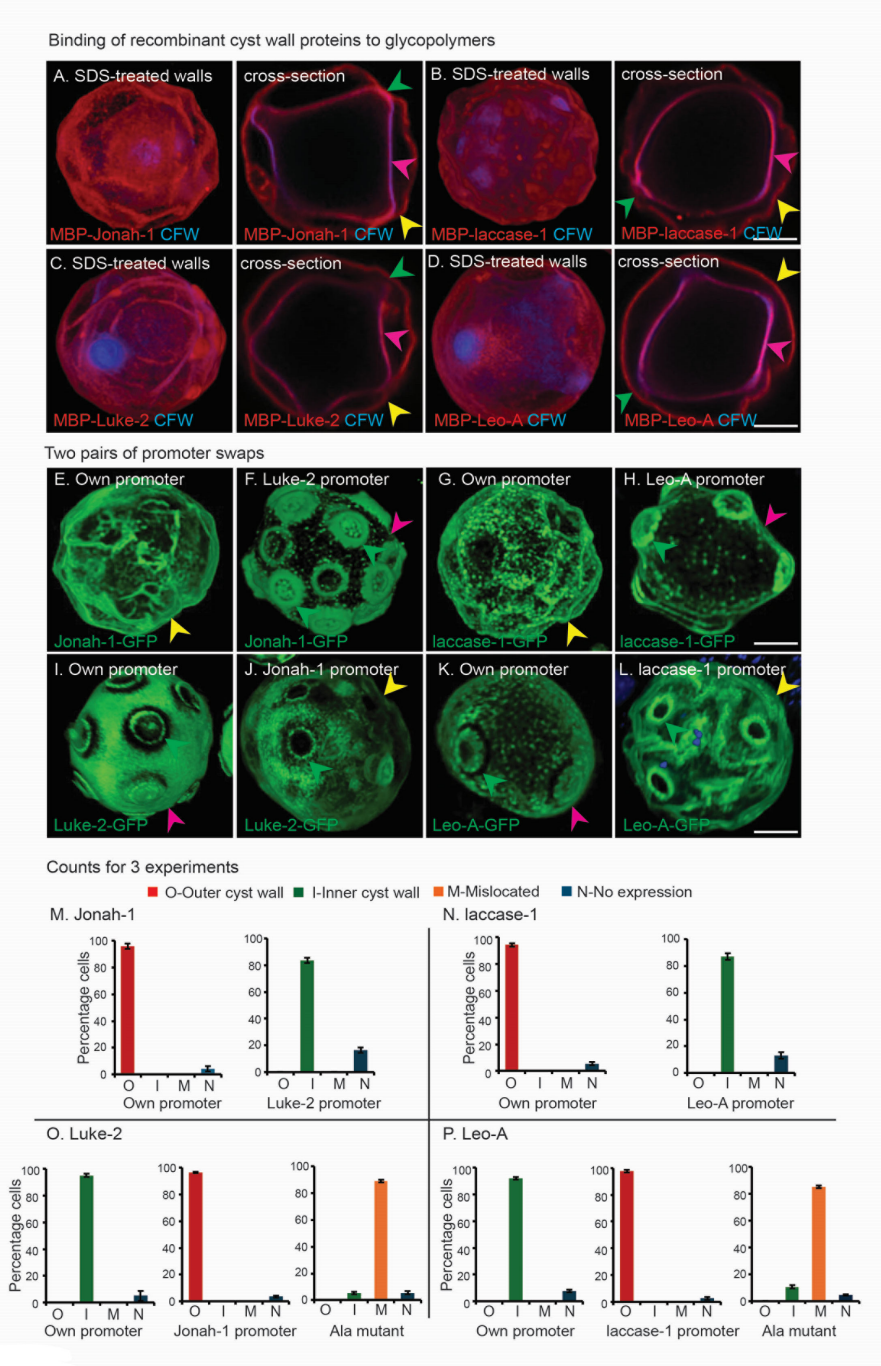
Localization of cyst wall proteins in the ectocyst layer, endocyst layer, and/or ostioles is for the most part determined by timing of expression during encystation. Deproteinated cyst walls were incubated with cyst wall MBP fusion proteins conjugated to Alexa Fluor 647. (**A** to **D**) MBP fusions for the BHF of Jonah-1, the CuRO-1 of laccase-1, and the entire Luke-2 and Leo-A minus their signal peptides all bind to both the endocyst layer (pink arrow heads), ectocyst layer (yellow arrowheads), and ostioles (green arrowheads). (**E**) In the first promoter swap, Jonah-1-GFP expressed under its own early promoter localizes to the ectocyst layer (see also Fig. 1B). (**F**) Expression under the later Luke-2 promoter causes Jonah-1-GFP to localize to the ostioles, which increase in thickness and number, while there is minimal localization of Jonah-1-GFP to the endocyst layer. (**I**) Conversely, Luke-2-GFP under its own later promoter localizes to the endocyst layer and forms a narrow ring around ostioles (see also Fig. 2B). (**J**) Expression under the early Jonah-1 promoter causes Luke-2 to relocate to the ectocyst layer. Remarkably, the narrow ring of Luke-2-GFP around the ostioles appears the same under either its own or the Jonah-1 promoter, suggesting its target glycopolymer is available early and later during encystation. (**G**) In the second promoter swap, laccase-1-GFP expressed under its own early promoter localizes to the ectocyst layer (see also Fig. 4D). (**H**) Expression under the later Leo-A promoter causes laccase-1-GFP to localize to the ostioles with minimal localization to the endocyst layer. (**K**) Conversely, Leo-A-GFP under its own later promoter localizes to the endocyst layer and forms a narrow ring around ostioles. (**L**) Expression under the early laccase-1 promoter causes Leo-A to localize to the ectocyst layer and forms a narrow ring around ostioles. These experiments show that under both early and later promoters, Jonah-1 and laccase-1 have similar localizations in the cyst wall, suggesting that each bind to the same glycopolymer(s). Luke-2 and Leo-A also have similar localizations under early and later promoters, but the localization is distinct from that of Jonah-1 and laccase-1, suggesting pairs of wall proteins are binding to different glycopolymers. Scale bars for panels A to L are each 5 µm. Manual counts carried out on transgenic cysts for GFP tagged protein localization to the outer cyst wall (red bar), inner cyst wall (green bar), mis-localized (orange bar) or no expression (blue bar). (**M**) Jonah-1-GFP under own promoter showing 93% of the cysts have protein localized to the outer cyst wall, and 84% of cysts under Luke-2 later promoter localizing to the inner cyst wall. (**N**) Laccase-1-GFP under own promoter showing 94% of the cysts have protein localized to the outer cyst wall, and under Leo-A later promoter, 86% of the cysts have protein localized to the inner cyst wall. (**O**) Luke-2-GFP under own promoter shows 94% of cysts have protein localized to the inner cyst wall, 96% to the outer cyst wall under Jonah-1 promoter, and 89% mis-localized in ala-mutated cysts. (**P**) Leo-A-GFP under own promoter shows 92% of cysts have protein localized to the inner cyst wall, 97% to the outer cyst wall under laccase-1 promoter, and 89% mis-localized in ala-mutated cysts. The counting was done three times, and a representative graph was plotted with mean ± SD.

### Unique sets of four disulfide knots of Leo lectins also contain linear arrays of three aromatic amino acids, which bind cellulose and direct the protein to the endocyst layer and ostioles

Leo-A (ACA1_074730) has two adjacent domains with eight Cys residues, while Leo-S(c) (ACA1_188350), which was corrected using a new, nearly completed transcriptome of encysting Ac, has two 8-Cys domains separated by a Thr-rich spacer (25–27). Structure predictions showed Leo-A and Leo- S(c), each containing two sets of four disulfide knots (4DKs), which link a series of short loops (Fig. 2A through D) (30–32). Structure-based searches with the 4DKs of Leo gave no hits, strongly suggesting the domain is unique to and was made by Ac. The 4DKs of Leo-A contain linear arrays of three aromatic residues (Y46, Y63, and Y77 and Y134, Y151, and Y165), which when mutated to Ala caused an MBP-Leo-A fusion to no longer bind to Avicel cellulose (Fig. 1H), and Leo-A-GFP to no longer localized to the endocyst layer and ostioles (Fig. 2E and F and 5P). These results suggest that the localization of Leo-A in the endocyst layer and ostioles is also dependent upon its binding to cellulose (and possibly chitin).

In a recent paper, we showed that Luke-2 (previous section) and Leo-A (here) are each made later in encystation and localize to the endocyst layer and ostioles (27). In contrast, after 12 h of encystation, Leo-S(c)-GFP is present in a dense set of secretory vesicles that fill the cytosol of cells, which are rounded but lack a wall (as shown by the failure to label with WGA or CFW) (Fig. 2G). After 24 h of encystation, Leo-S(c)-GFP forms a patchy distribution in a single-layered wall, which labels with WGA and CFW (Fig. 2H), while after 72 h, Leo-S(c)-GFP has a homogeneous distribution in the ectocyst layer of mature cyst walls (Fig. 2I). These results show Leo- S(c) is made early in encystation and localizes to the ectocyst layer, in contrast to Leo-A that is made later and localizes to the endocyst layer and ostioles (27).

### Jonah lectins, which have one or three three-sided β-helical folds, are made early in encystation and localize to the ectocyst layer

While Jonah-1 (ACA1_164810) contains a signal peptide, a Thr-rich domain, and a choice of anchor A (CAA) domain, Jonah-3 (ACA1_157320) predicted by AmoebaDB contains three CAAs and 12 transmembrane helices (TMHs) (Fig. 3A) (25–27, 45, 46). As no other Ac cyst wall protein identified to date contains a predicted TMH, we checked Jonah-3 versus the new transcriptome and found that corrected Jonah-3(c) contains a signal peptide and no TMHs (47, 48). Structure predictions showed that Jonah-1 contains a single BHF, two α-helices of unknown function, and a long unstructured domain rich in Thr, while Jonah- 3(c) contains three BHFs separated by long unstructured domains rich in Ser (Fig. 3B and C) (30–32). An end view shows that the Jonah-1 BHF is three-sided rather than two-sided, while a side view shows that the BHF is like that of the Antarctic bacterium *Colwellia* sp., the structure of which has been solved (PDB 3WP9) (Fig. 3D and E) (49, 50). The single BHF of Jonah-1 was fused to MBP for studies of its binding to deproteinated cyst walls (see below).

Confocal microscopy showed that Jonah-3(i)–GFP, which was synthesized from Twist Biosciences based on the incorrect sequence predicted by AmoebaDB, forms rings around ostioles (Fig. 3F). In contrast, Jonah-3(c)–GFP localizes to the ectocyst layer, which is the same place as Jonah-1 (Fig. 3G and J). Double labels show that Jonah-1–mCherry after 12 h of encystation is abundant in secretory vesicles, while Luke-2–GFP is not yet made (Fig. 3H) (51). After 24 h of encystation, Jonah-1–mCherry is present in the single-layered wall, while Luke-2–GFP is in secretory vesicles (Fig. 3I). After 48 h of encystation, Jonah-1 is in the ectocyst layer, and Luke-2 is in the endocyst layer and ostioles, which are the final locations for each protein in mature walls (Fig. 3J). These double labels justify the first promoter swap between Jonah-1 (early) and Luke-2 (later) (see below).

### An abundant laccase, which has three cupredoxin-like domains, is also made early in encystation and localizes to the ectocyst layer

Structure predictions showed that Ac laccase-1 (ACA1_068450), which is abundant in purified walls, has three cupredoxin-like domains (CuRO-1, CuRO-2, and CuRO-3) (Fig. 4A and B) (25–27, 52–54). Remarkably, the laccase of *Melanocarpus albomyces* binds cellulose. The CuRO-1 of laccase-1, which was fused to MBP to study its binding to deproteinated cyst walls (see below), closely resembles CuRO-1 of the spore coat protein A of *B. subtilis*, the structure of which has been solved (PDB 4YVN) (Fig. 4C).

GFP-tagged laccase-1 fills secretory vesicles of Ac encysting for 18 h when there is no wall labeling with WGA or CFW (Fig. 4E). After 24 h of encystation, laccase-1-GFP is homogeneously distributed in the single-layered wall (orange arrowheads), which becomes the ectocyst layer (yellow arrowheads) of mature cysts at 72 h (Fig. 4E and F). Double labels show that laccase-1 tagged with red fluorescent protein (RFP) is abundant at 18 h of encystation in secretory vesicles before Leo-A–GFP or cyst walls are made (Fig. 4G). After 24 h of encystation, laccase-1-RFP is in the single-layered wall, while Leo-A-GFP is in secretory vesicles (Fig. 4H). After 72 h (mature cysts), laccase-1-RFP is in the ectocyst layer, while Leo-A–GFP outlines the ostioles and lightly labels the endocyst layer (Fig. 4I). These double labels justify the second promoter swap (next section) between laccase-1 (early) and Leo-A (later).

### Binding of Luke, Leo, Jonah, and laccase to deproteinated walls and promoter swaps show timing of expression is the major determinant of localization in the ectocyst layer (early) or endocyst layer and ostioles (later)

When wall proteins were removed with SDS and proteinase K, CFW labeling showed Ac cyst walls still have ectocyst and endocyst layers, which are connected by ostioles that are best visualized in 2D cross-sections (Fig. 5A through D). To our surprise, MBP-fusions to the BHF of Jonah-1, CuRO-1 of laccase-1, and the entire Luke-2 and Leo-A minus their signal peptides all bound in a similar pattern to both layers of the cyst wall, suggesting that cellulose and/or chitin, to which each recombinant wall protein binds, are present in both layers (22, 24, 27, 41, 42).

In the first promoter swap, Jonah-1-GFP, expressed under the later Luke-2 promoter, moves from the ectocyst layer to the ostioles, which are densely coated, while the endocyst layer is weakly labeled (Fig. 5E and F). Conversely, Luke-2–GFP, expressed under the early Jonah-1 promoter, moves from the endocyst layer and ostioles to lightly decorate the ectocyst layer and form flat, narrow rings around the ostioles (Fig. 5I and J). These narrow rings, which match those of Luke-2-GFP under its own promoter, suggest that the glycopolymer(s) in the ostiole, to which Luke-2 binds, is present both early and later during encystation. Quantitation of three repeats of these promoter swaps and those in the next section shows that individual images of GFP-tagged proteins are representative of the group, with the exception that some cells have too weak a signal to judge localization (Fig. 5M through P). The latter is a common artifact of expressing GFP-tagged proteins on episomal plasmids, which may vary wildly in number within a set of transfected cells, even when G418 selection is maintained.

In the second promoter swap experiment, laccase-1-GFP expressed under the later Leo-A promoter moves from the ectocyst layer to the ostioles, which are densely coated (Fig. 5G and H). Leo-A–GFP, expressed under the early laccase-1 promoter, moves from the endocyst layer and ostioles to decorate lightly the ectocyst layer and form flat rings around the ostioles (Fig. 5K and L). Again, counts show that individual images are representative of the group (Fig. 5N and P). Promoter swaps then supports the idea that early expression does not just correlate with but causes proteins to localize to the ectocyst layer, and later expression causes proteins to localize to the endocyst layer and ostioles.

### How BJRFs of Luke, 4DKs of Leo, and BHFs are duplicated to make multidomain proteins, which are then increased in copy number, is different for each family of wall proteins

**Fig 6:**
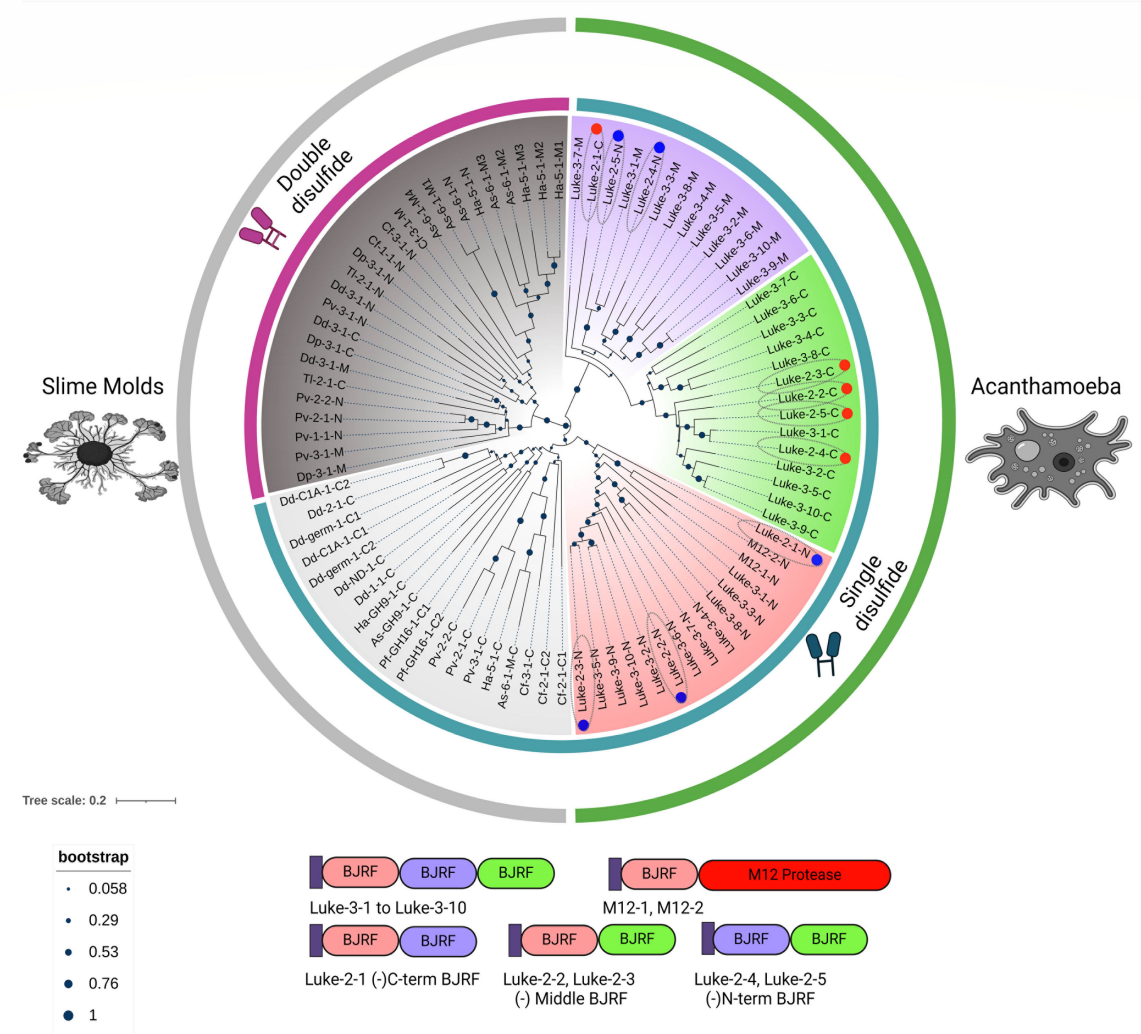
A neighbor-joining tree with bootstraps marked reveals common ancestry of N-terminal BJRFs of Luke and C-terminal BJRFs of slime molds and shows Luke-2s are truncated versions of Luke-3. A phylogenetic tree contains BJRFs of Ac (outer green boarder) or slime molds (outer gray boarder). BJRFs of Luke lectins, Ac M12 proteases, and some BJRFs of slime molds contain a single disulfide knot (inner blue-green boarder), while other BJRFs of slime molds have two disulfide knots (inner red boarder). See File S3 for their sequences. BJRFs of bacterial and plant endocellulases, which have no disulfides and are much less like BJRFs of Ac and slime molds, were not included in the tree. N-terminal, middle, and C-terminal BJRFs of 10 Luke-3s form three distinct clades marked in orange, purple, and green, respectively, while N- and C-terminal BJRFs of five Luke-2s are marked with blue and red circles, respectively. C-terminal BJRFs of 19 slime mold wall proteins and enzymes form a clade marked in light gray, while N-terminal and middle BJRFs of slime mold wall proteins form a clade marked in dark gray. Although the bootstrap support is modest (0.53), clades BJRFs at N-termini of Luke lectins and C-termini of slime mold proteins, each of which have a single disulfide, appear to share common ancestry. In contrast, common ancestry of clades of middle and C-termini BJRFs of Luke has strong bootstrap support (1), and common ancestry of the clade of BJRFs of slime molds with two disulfides has strong bootstrap support (0.76). Cartoons of domains of Luke-2s show they are missing either the N-terminal BJRF (Luke-2-4 and Luke-2-5), middle BJRF (Luke-2-2 and Luke-2-3), or C-terminal BJRF (Luke-2-1).

A BJRF phylogenetic tree of five Luke-2s, 10 Luke-3s, and two M12 metalloproteases, as well as 14 wall proteins and six enzymes of slime molds, which was made by the neighbor-joining method because the sets were so large, revealed the following (Fig. 6) (55–57). First, Ac BJRFs, all of which have a single disulfide linking the beginning and end of the domain, form three distinct clades that group N-terminal, middle, and C-terminal BJRFs of Luke-3s. Slime mold BJRFs form one clade with a single disulfide, which groups C-terminal BJRFs of wall proteins and enzymes, and a second clade with two disulfides, which groups N-terminal and middle BJRFs of wall proteins. Second, the N-terminal BJRFs of Ac are more closely related to C-terminal BJRFs of slime molds than to middle and C-terminal BJRFs of Ac, suggesting the former share common ancestry in Amoebozoa, while the latter BJRFs evolved independently in Ac. Similarly, BJRFs with two disulfides evolved independently in slime molds. Third, Luke-2s evolved from Luke-3s by the loss of the C-terminal BJRF (Luke-2-1), loss of the middle BJRF (Luke-2-2 and Luke-2-3), or loss of the N-terminal BJRF (Luke-2-4 and Luke-2-5) (Fig. 6).

**Fig 7:**
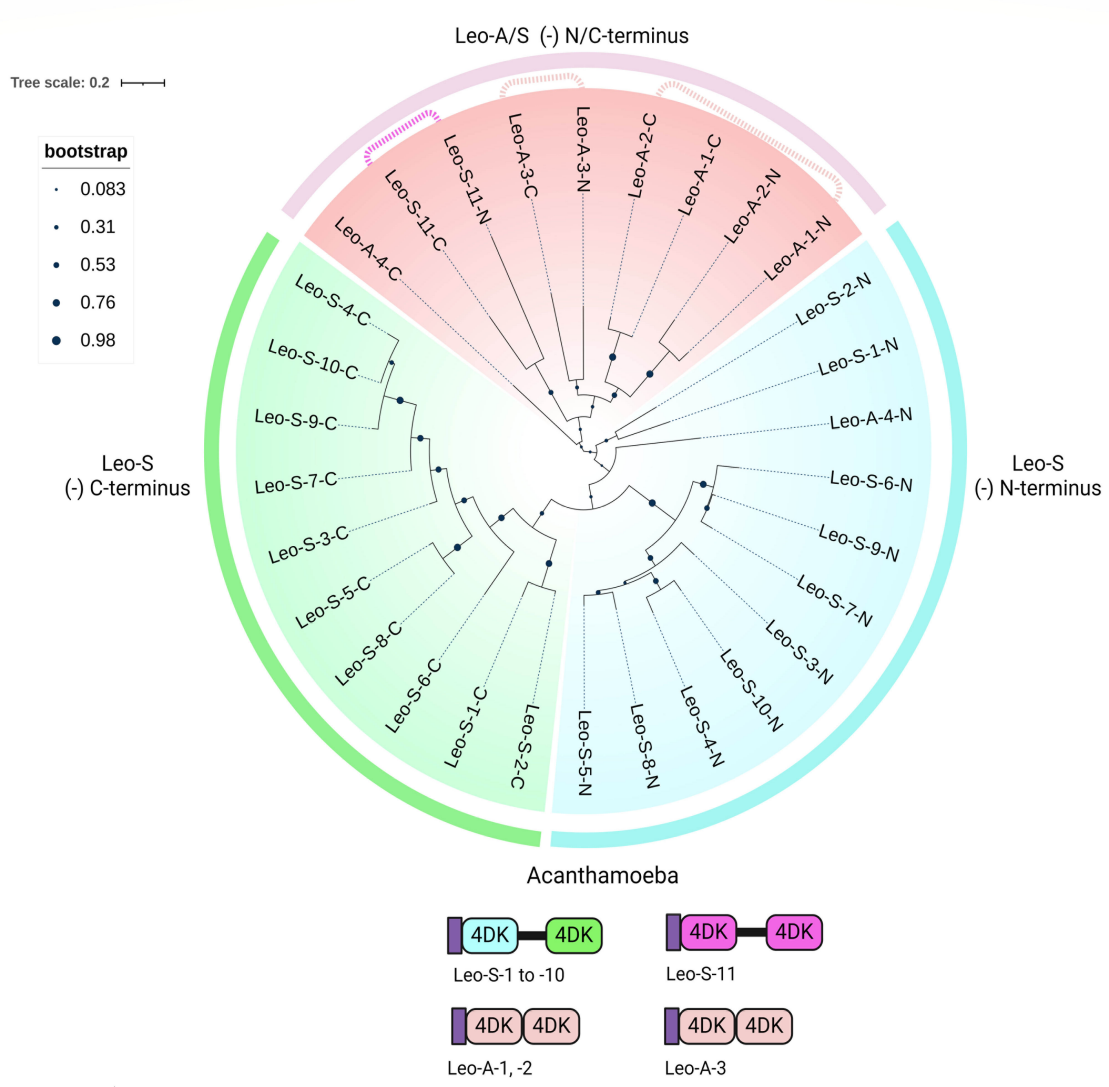
A maximum likelihood tree (JTT matrix-based model) shows one copy of Leo-S with a long Ser-rich spacer between 4DKs, which are unique to Ac, has been duplicated nine times, while one Leo-A with adjacent 4DKs has been duplicated once, and two other Leo-As are single copy. A phylogenetic tree contains 4DKs of Leo lectins, which are unique to Ac. See File S3 for their sequences. One clade, which has reasonable bootstrap support (0.76), includes N-terminal 4DKs (blue) of 10 Leo-Ss and a single Leo-A, while another clade with bootstrap support includes C-terminal 4DKs (green) of 10 Leo-Ss. This result suggests that the spacer was added between 4DKs, and then the genes were duplicated nine times. In both clades, Leo-S-1 and Leo-S-2 look more like each other than Leo-S-3 to Leo-S-10, suggesting their genes were duplicated most recently. Leo-As with adjacent 4DKs (orange) appear to have been made independently and replicated once (Leo-A-1 and Leo-A-2) or not replicated (Leo-A-3). Finally, Leo-S-11 has no relationship to the other Leo-Ss or Leo-As, suggesting its origin is unique.

A phylogenetic tree of 4DKs, which was made by the maximum likelihood method, including four Leo-As and 11 Leo-Ss, revealed the following (Fig. 7). First, N-terminal and C-terminal 4DKs of Leo-S-1 to Leo-S-10 form separate clades, which is consistent with the idea that the Thr-rich spacer was added once, and then nine paralogs were made. Second, Leo-As seems to have been made independently three times when a single 4DK was duplicated (Fig. 7). Whether the Leo-A derives from Leo-S, as suggested by the similarity of 4DKs of Leo-A-4-N and Leo-S-1 and -2, or from a single 4DK, which is no longer present in the Ac genome, is not clear.

**Fig 8:**
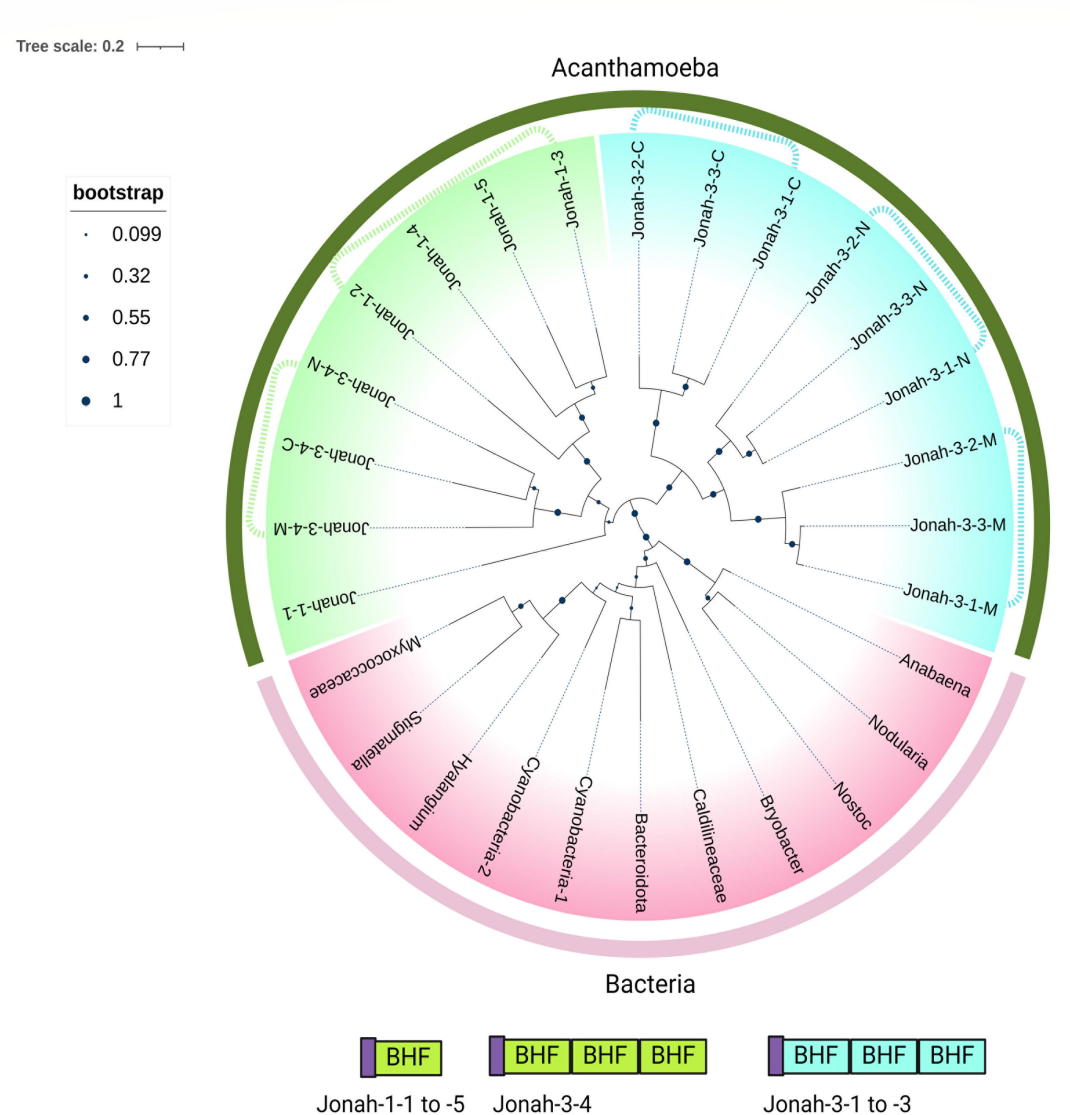
A maximum likelihood tree (JTT matrix-based model) shows BHFs of Jonah, which were derived from bacteria by HGT a long time ago, are stable as a single copy in five Jonah1s, while one Jonah-3 with three BHFs was duplicated twice, and in the other Jonah-29, three has not been duplicated. A phylogenetic tree shows that BHFs of Jonah lectins (dark green border) are distinct from those of bacteria (pink border), so that it is not clear from which bacterium the Ac genes are derived by HGT. See File S3 for their sequences. BHFs of five Jonah-1s are in the same clade as N-terminal, middle, and C-terminal BHFs of Jonah-3-4 (yellow), suggesting they share common ancestry, and each gene is stable. N-terminal, middle, and C-terminal BHFs of Jonah-3-1 to Jonah-3-3 are in the same clade (green), suggesting a single Jonah-3 with three BHFs was duplicated twice.

A BHF phylogenetic tree of five Jonah-1s and four Jonah-3s and 11 bacterial proteins, which was made by the maximum likelihood method, revealed the following (Fig. 8). First, the clade of Jonah BHFs is distinct from the clade of bacterial BHFs, suggesting horizontal gene transfer (HGT) happened a long time ago, so the precise bacterial donor cannot be identified. Second, BHFs of Jonah-1s have long branch lengths, suggesting paralogs were also made a long time ago and are stable in the Neff genome. Third, Jonah-3s appear to have been assembled on two occasions: one gene encoding a protein with distinct N-terminal, middle, and C-terminal BHFs was duplicated twice to make three paralogs (Jonah-3-1, -3-2, and -3-3), while the gene for Jonah-3-4 was not duplicated (Fig. 8).

## DISCUSSION

### Luke and Jonah lectins derive from bacteria and plants by lateral gene transfer

Luke lectins have two or three BJRFs, which share recent ancestry with wall proteins of *Dictyostelium* and distant ancestry with CBM2 and CBM49 of bacterial and plant endocellulases (33, 38, 40, 56, 57). The similarity between BHFs of Jonah lectins to ice-binding proteins of Antarctic bacteria may explain how *Acanthamoebae* are able to survive in frozen lakes in Norway and the Antarctic (49, 50, 58, 59). That said, it is not easy to understand why Jonah and its BHFs have not been lost from Ac, which, for the most part, live in warm soil and water that are increasing with climate change (13). Laccases, which are most like those of bacteria based on a phylogenetic analysis that will be shown elsewhere, are widely distributed in walls of plants, fungi, and oomycetes (52–54). Similarly, the substrate and activity of the laccases will be determined in separate experiments.

### While its 4DKs that bind cellulose are unique, Leo lectins share properties with wall proteins of Ac and other organisms, suggesting the importance of convergent evolution

Chitin-binding domains of *Saccharomyces* chitinases (CBM19), *Entamoeba* chitinases and Jessie lectins (CBM55), and WGA (CBM18) also contain distinct 4DKs, while chitin-binding domains of *Entamoeba* Jacob lectins contain 3DKs (34, 37, 60–63). The 4DKs of Leo and BJRFs of Luke each contain linear arrays of three aromatic amino acids, which Ala mutations showed bind cellulose. Similar linear arrays of three aromatics that bind cellulose are present in endocellulases of bacteria (CBM2) and plants (CBM49), as well as in CBM63s of expansins (proteins that unfold cellulose) of plants, bacteria, and Ac (not shown here) (33, 35, 38). The reinvention of these linear arrays of three aromatics in three sets of unrelated structures strongly suggests they are an excellent means for binding cellulose.

Like Leo-A with two adjacent 4DKs and Leo-S with 4DKs separated by a large Thr-rich spacer, *Entamoeba* Jacob-1 has two adjacent 3DKs, while Jacob-2 has a third 3DK separated by a long, unstructured, Ser-rich spacer (64). Unstructured Ser- or Thr-rich domains, which are also present in Jonah and Luke lectins, are likely modified by *O*-linked glycans, which protect glycoproteins from being degraded by bacterial proteases, as shown for wall proteins of *Entamoeba* and *Cryptosporidium* (65, 66). We did not identify *O*-linked glycans on Ac cyst wall proteins, nor did we confirm the AlphaFold structures by crystallizing any of the four wall proteins studied here. This seems less of an issue for the BJRFs of Luke, BHFs of Jonah, and copper oxidase domains of laccase, the structures of which match those of crystallized proteins (40, 49, 54). A goal of future studies will be to solve the crystal structure of the unique 4DK of Leo +/− cellulose.

Timing of expression appears to be the major determinant for localization in the ectocyst layer, endocyst layer, and/or ostioles. Here, we increased the number of ectocyst layer proteins from just Jonah-1 to four with the addition of Jonah- 3(c), Leo-S(c), and laccase-1, which may be targets for antibodies to diagnose Ac cysts in eye smears of patients with AK (1–3). Laccase-1 and Leo-S(c) were also localized to a large set of secretory vesicles early in encystation, as was previously shown for Jonah-1 (27).

For the first time, we used double labels to colocalize Jonah-1 and Luke-2 or laccase-1 and Leo-A in developing walls to show unequivocally that the ectocyst layer is made early, and the endocyst layer and ostioles are made later. Promoter swaps showed Jonah-1 and laccase-1 switch to the endocyst layer and ostioles when expressed under later promoters of Luke-2 and Leo-A, respectively. Conversely, Luke-2 and Leo-A switch from the endocyst layer and ostioles to the ectocyst layer when expressed under the early promoters of Jonah-1 and laccase-1, respectively. Because the localizations of Jonah-1 and laccase-1 resemble each other under early and later promoters, it is likely that they are binding to the same glycopolymer(s), which we did not identify here. Similar localizations of Luke-2 and Leo-A under both promoters suggest these lectins are also binding to the same glycopolymer(s), which is not surprising, as Luke-2 and Leo-A contain similar arrays of three aromatic amino acids that bind cellulose.

### Domain duplication, which is obligate for Luke and Leo and optional for Jonah, occurs by flexible methods, resulting in multiple forms of each cyst wall protein

While Jacob and Jessie lectins in the cyst wall of *Entamoeba* and oocyst wall proteins of *Cryptosporidium* (COWPs) and *Toxoplasma* (TgOWPs) have been shown to be in multiple copies with varying numbers of duplicated domains, this is, to our knowledge, the first time that phylogenetic trees have been used to determine the origins and evolution of duplicated domains in wall proteins of a pathogen (67, 68). Three BJRFs are present in the ancestral Luke-3 lectin, which was duplicated at least 16 times and then truncated to two BJRFs five times. The single BJRF at the beginning of two M12-proteases suggests the possibility that the enzymes are targeted to the cyst wall and/or are glycopeptidases, as has been shown for some bacterial metalloproteases (69, 70). The ancestral Leo-S with two 4DKs separated by an unstructured, Thr-rich spacer was duplicated at least 10 times in the Ac genome. It is not clear whether multiple Leo-As, which lack a spacer, resulted from duplications of a single 4DK or from duplication of the N- or C-terminal 4DK of Leo-S. Finally, Jonah lectins are stable with one or three BHFs, while Ac laccases have three copper oxidase domains required for enzyme activity, which was not determined here (54). Although there are numerous aromatic amino acids on the surfaces of Jonah-1 and laccase-1, none are in linear arrays, so we did not attempt to make Ala mutants to test cellulose-binding sites.

### Protein prediction by AmoebaDB is unreliable

While Ac protein sequences available in AmoebaDB made it possible for us to perform mass spectrometry of cyst walls and for others to discover dozens of proteins of interest, its predictions are based upon an incomplete genome of Neff strain of Ac and incomplete transcriptomes (25–27, 71). We used a new, nearly complete transcriptome to show 12 TMHs between three BHFs, which incorrectly localized Jonah-3-GFP to a ring around ostioles, were caused by frameshifts. The new transcriptome also allowed us to correct an eight-amino acid-long deletion in Leo-S that would have disabled its N-terminal 4DK, which is necessary for binding cellulose and localizing the protein to the ectocyst layer. For the phylogenetic trees, sequences of four Luke BJRFs, two other Leo 4DKs, and two other Jonah BHFs were corrected using the new transcriptome, while three BJRFs, one 4DK, and three BHFs, which were absent in AmoebaDB, were confirmed in the 2021 Ac genome by TBLASTN (72). These errors are likely the tip of the iceberg for the 14,000+ proteins predicted by AmoebaDB.

## MATERIALS AND METHODS

### Summary of new methods to study Ac wall proteins

Many methods that we used in our mass spectrometric characterization of proteins in purified cyst walls of Ac are the same, which were described in detail (27). A new, nearly complete transcriptome, which will be described elsewhere, was used to fix incorrect protein predictions by AmoebaDB, as well as identify missing proteins for phylogenetic trees. AlphaFold was used to predict structures for each cyst wall protein and test with Ala mutations aromatic amino acids of Luke and Leo involved in binding cellulose. Double labels with GFP and either RFP or mCherry were used to compare the expression and localization of ectocyst and endocyst layer proteins in the same cell. Because SuperFect Transfection Reagent was discontinued, plasmids were transfected into Ac trophozoites with Lipofectamine. Glycopolymers were exposed in both layers of the cyst walls by removal of wall proteins using SDS and proteinase K. Two pairs of promoter swaps were used to show that localization in the two layers of the cyst wall is not just correlated with but is caused by timing of expression. Confocal microscopy replaced structured illumination microscopy (SIM) because it is quicker and allowed us to examine many more parasites. Finally, phylogenetic trees were used to determine the origin of duplicated domains in Luke, Leo, and Jonah lectins.

### *In silico* structural predictions of candidate cyst wall proteins

The 3D structure models of different cyst wall lectins were predicted by AlphaFold2 using the Colab server (30). The molecular visualization of active site residues, 3D structures, and structural superimposition were performed using PyMOL Molecular Graphics System, Version 2.0 Schrödinger, LLC (73). The predicted 3D structures were compared to AlphaFold and PDB database using Foldseek server (31). Other *in silico* tools used to functionally characterize the cyst wall proteins include the CDD database (conserved domain identification) (74), SignalP 4.1 (48) and DeepTMHMM (transmembrane helices) (47), CAZy and InterPro databases (carbohydrate-binding modules) (34, 37, 46), and big-PI (glycosylphosphatidylinositol anchors) (75). To test their role in binding cellulose, linear arrays of aromatic amino acids in BJRFs of Luke-2 and 4DKs of Leo-A, which were identified by AlphaFold, were mutated to Ala and expressed as MBP fusions in the periplasm of *E. coli*, as previously performed for wild-type Luke-2 and Leo-A (27, 41, 42). MBP fusions and MBP alone (negative control) were then incubated with Avicel cellulose, and bound proteins identified by Western blotting, using the same exact methods described in reference 27. Alternatively, MBP fusions, including the BHF of Jonah-1 and CuRO-1 of laccase-1, were labeled with Alexa Fluor 488 and incubated with Ac cyst walls deproteinated with 1% SDS and 1 mg/mL proteinase K for 16 h at 37°C temperature. Deproteinated cyst walls labeled with MBP fusions were imaged with Nikon Ni2 AX inverted microscope (described below). All the assays were carried out three times, and a representative image and/or Western blot is shown.

### Culture, encystation, and transfection of *Acanthamoebae*

Ac Neff strain (ATCC 30010) parasites were obtained from the American Type Culture Collection (ATCC) and used in all experiments. Ac trophozoites were grown and maintained in axenic culture at 30°C in T-75 tissue culture flasks in 10 mL of ATCC medium 712 (PYG plus additives) with antibiotics (Pen-Strep) (Sigma-Aldrich Corporation, St. Louis, MO), as described previously (14, 27). Cysts were prepared from trophozoites by incubating them with an encystation medium (EM; 20 mM Tris-HCl [pH 8.8], 100 mM KCl, 8 mM MgSO_4_, 0.4 mM CaCl_2_, and 1 mM NaHCO_3_) for 120 h (15, 27).

GFP-tagged proteins under their own promoter (400 to 600 bp 5′ to the start codon) were expressed in the pGAPDH vector, which has a neomycin resistance gene for G418 drug selection (27–29). The full-length coding sequences of each protein were codon optimized and custom synthesized from Twist Biosciences, Quincy, MA, along with their respective promoter sequences. Primers for making constructs are listed in Supporting Information S1, while sequences of promoters and proteins are listed in Supporting Information S2. For the promoter swap experiments, the Luke-2 promoter was replaced with Jonah-1, while the laccase-1 promoter was replaced with the Leo-A promoter and vice versa. For visualizing two proteins in the same cell, a single pGAPDH vector was engineered to express Jonah-1 tagged with RFP or mCherry and Luke-1- GFP, each expressed under its own promoter (51). For construct preparation, we used NEBuilder HiFi DNA assembly master Mix from New England Biolabs, Ipswich, MA. Final constructs were confirmed using Oxford nanopore sequencing technology from Plasmidsaurus, Eugene, OR. Transfection of Ac was performed using Lipofectamine Transfection Reagent (Thermo Fisher Scientific), as per the manufacturer’s instructions. After 24 h of transfection, the medium was replaced with fresh media containing G418 (12.5 µg/mL). After 48 h of transfection, cells were transferred from six-well plates to T-75 flasks with ATCC medium 712 plus G418 (25 µg/mL).

### Confocal microscopy

Transgenic Ac trophozoites and encysting organisms were fixed with 4% paraformaldehyde for 15 min at room temperature, stained with 100 μg/mL of WGA (Thermo Fisher Scientific) conjugated to Alexa Fluor 647 and 50 μg/mL of CFW (Sigma Aldrich), washed, and mounted in VECTASHIELD Antifade Mounting Medium (Vector Laboratories, Newark, CA) (27). Samples were illuminated using 380-nm (CFW), 488-nm (GFP chimera), and 647-nm (WGA or RFP and mCherry chimeras) laser excitation. The fluorescence images were captured using CFI Plan Apochromat VC 60XC NA 1.42 oil objective of Nikon Ni2 AX inverted confocal microscope equipped with FX-Format F-mount cameras Digital Sight 10 and Digital Sight 50M. We deconvolved 0.1-µm optical sections using NIS elements (Version: AR5.41.02) imaging software. All confocal images shown were 3D reconstructions using dozens of z-stacks. Size bars were based upon 2D cross-sections.

### Quantification of microscopy

To check the homogeneity of the transfected lines, all the transgenic lines were encysted as mentioned above. The cells were fixed and stained as mentioned above and counted manually for GFP-tagged protein localization to the outer cyst wall, inner cyst wall, mis-localized, or no expression. The counting was done three times, and a representative graph was plotted with mean ± SD.

### Phylogenetic trees

Amino acid sequences of wall proteins were retrieved from AmoebaDB or the new transcriptome. BJRFs of Luke, 4DKs of Leo, and BHFs of Jonah, each of which has Cys residues at N- and C-termini, were named for parent protein and relative position (e.g., Leo-2.1-N) (see File S3). BLASTP was used to identify similar proteins from other species in the NR database at NCBI (45). Multiple sequence alignments were made using the Muscle, and MEGA-X was used to make neighbor-joining trees with 1,000 bootstrap replicates (55, 76). The trees were further colored and visualized using the iTOL (interactive tree of life) online tool (77).

The evolutionary history was inferred using the maximum likelihood method based on the JTT matrix-based model.

## SUPPLEMENTAL MATERIAL

File S1 msphere.00466-24-s0001.xlsx Primers used for making constructs.

File S2 msphere.00466-24-s0002.xlsx Promoter sequences and coding sequences used for making GFP-tagged proteins and MBP fusions.

File S3 msphere.00466-24-s0003.docx Sequence used for constructing the phylogenetic tree.

ASM does not own the copyrights to Supplemental Material that may be linked to, or accessed through, an article. The authors have granted ASM a non-exclusive, world-wide license to publish the Supplemental Material files. Please contact the corresponding author directly for reuse.
