# Supplementary material for: Cellulose binding and the timing of expression influence protein targeting to the double-layered cyst wall of Acanthamoeba
Source: mSphere. 2024 Aug 13;9(9):e00466-24. doi: 10.1128/msphere.00466-24 (PMC11423589; doi:10.1128/msphere.00466-24)
Supplement: File S3 — Sequence used for constructing the phylogenetic tree. [file msphere.00466-24-s0003.docx]

S1 word file: Sequences used for phylogenetic trees of Luke BJRFs, Leo 4DKs, and Jonah BHFs.

BJRFs of Luke lectins and wall proteins and enzymes of slime molds

>Luke-2-1-N ACA1_377670

CTASLNQTRQASWVDSEQFPRSLWTVEIRNTGQQAVTNVLLSIQGSINQIWEVVLVNDSLYRLPDWRLQVGGIPAGQSHVFGFIVNNNSTAAPVSLVSVQC

>Luke-2-1-C

CSLLASQVARSGAGGSWSDNSNRFQIYDITLNNNGASRLTQASLTIAIAADQAIAQFWNLERVDATNTFNIESFLLPAPGGSQSGLGYVLQTPLNSTSDGSSIGAVNFRC

>Luke-2-2-N ACA1_270280

CSVSVQQTLGSAWKQDGVDMSQWNAQLTAGSAAVKSVQLAITGAGGFTQLWELTRTSAGVYVLPDYRLQSGGLPAGQVHHFGYIWKSASPATITVASIDC

>Luke-2-2-C

CSAIVELTARSAANGGVWTDAAGRTYQIFQVLITNRGQRPVNGGVITFDLVNGAEISQYWELNRQGPTSPAFNIAFNYGQLQPGASQGAGVVVSSAAKPSFTLSSVAC

>Luke-2-3-N Neff_2021-tblastn

CNVSIKQTLGSAWQQEGKSLSQWNVQLTAGSEAVKSLELSITGAKIIQFWELTNQSTGLFALPEYRIQNGGIVAGQVHQFGYIWESSAQATITVASINC

>Luke-2-3-C

CGASVSISTRTRSGGGVWQENGSTFQIFDLTITNNGQRAVNGGVVTFGLVEAGAAITQSWELSRQGSTSNAFNVAFGYGPLQPGASQGAGIVVRLAGSTSAAQPTVTLSGLAC

>Luke-2-4-N ACA1_246110(c)

CQLSVSQITRSGGSWTQGDFFFQIYDLPIVNTGSSPVTSAVIAIDTAPNQEQVITQFWNLARQSATSNLFNVANPGGNIEVGATLGAGYIVRTPLSAGSQVPPTTRLVSVNC

>Luke-2-4-C

CNAAVSIVARSAAAGGSWTTGPNQFFQIFDITITNTGQRPLNGGVLTFGLPVAGSTITQWWELNRQGNTNVFNVAFNFGPLLVGASQGAGIVVQTSSPSQALPSAVLSNLAC

>Luke-2-5-N ACA1_096300

CQLSVPQLTRPGGSWTEGDFRFQIYDLPIVNEGSRPVTAAVVSINLASSQDQMITQFWNLQRQSSTSNLWNVVNPGGNIEVGSTLNSGFVVRSPISAGPQVAPVTTLVSVSC

>Luke-2-5-C

CSAVVASIVPRSAAAGGNWSSGPNQFFQIFDILITNNGERPVNGGVITFGLPVAGSSISQSWELNRQGNSNVFNVAFNYGPLQVGASQGAGIVVQTSSPSQAVPSAQLSNITC

>Luke-3-1-N ACA1_245650

CAVRIEQSLGSAWNGAGGVPMSQWNARIVSSGSETVTALQVAFGAPTTAIDQVWGLEPVAGASRVYDLPDYVILNGGLASGQAFNWGYIWESSAQAPLSVASVQC

>Luke-3-1-M

CQLSVSQTTRPSSAGGSWTEGDFFFQIYDLTLLNSGSRPVSSAVIAIDTTSNRQQVITQFWNLERQSATSDLFNVRNPGGNIEVGATLGAGYIVRTPLSAGSQVPPTTRLVSVNC

>Luke-3-1-C

CNAAVSIVARSAAAGGSWTTGPNQFFQIFDITIANTGQRPLNGGVLTFGLPVAGSTITQWWELNRQGNTNVFNVAFNFGALQVGASQGAGIVVQTSSPSQVLPSAVLSNLAC

>Luke-3-2-N ACA1_187760(c)

CSVSIKQTLGSSWKQDGKDMSQWNVQLTAGSETVKSLELSITGAALTQVWELTRQPNGLFALPDYRLQSGGIVAGQVHQFGYIWESAAQATITVASINC

>Luke-3-2-M

CSVSVTQTARSANAGGSWTDGAYRFQLYDLAIVNNGNSPVTAAQLTIALAAGQAITQSWNIELKSGTASTYVVPNTYGPLQVGATLGAGYVLRSPLAAGQPAAPAIAIDSVTC

>Luke-3-2-C

CGAVVTITARSAAAGGVWSDNGNTFQIFDVNLANSGQRPINGGVLTFAFSEASATITQFWELNRHGATNAFNVAFNYGPLQVGAIQGSGIIVRVSGSSPAAKPSTTLSSLSC

>Luke-3-3-N ACA1_252830

CKVDIVQTLGSAWTVNGQAYSQWSVTITAGPKDVKSLTLSISGNFDQLWEITKDFRGLYVLPDYVLQGGGIHAGKTHNFGYIIKSQSKAIIDIELVDC

>Luke-3-3-M

CKVTTQQTARSAAEGGQWKDATYTYQIYDLAVKNAGDKVVTTAQLTLTLASGASFYQFWNLERKEASETFFVP

NYGGIQVGATQTAGYVVRTPLTNGAPTAPIVHVDSTTC

>Luke-3-3-C

CSAKVSIVARSGSNWQDASGFNQLYDITITNTGSRAINGGQVTFGLGSGVGVTQYWELNRKTANVFGIPTTYGPLQVGASQGAGIVATRNQAPIPLPTVTLDSVTC

>Luke-3-4-N ACA1_253500

CQVSVTQTLGSAWTTNGQDFSQWSVAITNTGSEGVKSVDLSIANDSLFDQLWNIVKDNRGLYVLPDYILQNGGIAVSGSYQFGYIIKSKAQATIDLEAVDC

>Luke-3-4-M

CTVTVESVARSAASGGQWTEGDSQFQIFDVTTTNSGNTPVTAVKLTISVSGADVSIYQFWNLERKENTNVFNVPTPWGAIQVGASQGAGFIVKSKEGQSVAAPVVKVESATC

>Luke-3-4-C

CSAELTIVARSAASGGQWQDGNKWNQIFDVVVSNTGSKALTGGQVSFTLASGVSVSQFWELNRKDATTFTIPTTYGPIQVGASQGAGIVATTTTATTIATPTATLSGLTC

>Luke-3-5-N ACA1_287530

CNVSVKQTLGSAWQQEGKNLSQWNVQLTAGSEAVKSLELSITGAKIIQLWELTNQSTGLFALPEYRIQNGGIVAGQTHQFGYIWESSSQATIAVSSINC

>Luke-3-5-M

CSVSVAQTTRSASAGGSWIDGAFHFQIFDLTFLNNGNQAVNNAQVTITLAAGQEITQSWNIERKSGSTFTTSTTYGPLQVGATLGAGYVLRSAASAGQPAAPAAPAVAIDSVTC

>Luke-3-5-C

CSASVTVVARSAAAGGVWVDGGNTFQIFDVSIANNGQRPLNGGVVTFTLAEAGATITQFWELSRQGSTNAFNIAFNYGALQAGATQGAGVVVRLAGSSPAAKPTVTIGSLGC

>Luke-3-6-N ACA1_061050

CNVSVKQTLGSAWKQNGQDMSQWNAQITAGSENVKSVVLSITGAKITQLWELTLDEATGLYSLPAYRLQSGGLSAGQTHQFGYIWESAAQATITVSSINC

>Luke-3-6-M

CSLTVTQTKRSAAEGGSWTTGDFGFQIYDLALANNGQRPANGARINVALVSGAQQQIYQFWNVERASADSTVFNVPTPWGSIQVGASQGAGYILRYPLSAAAQQAAPTTTLLSVTC

>Luke-3-6-C

CSATVSIAARSAANSGSWTANGANFQIFDLVISNTGSKALTDAQLTFALPDGTTVFQWWELMSIANQASVFDVSFNYGPLQVGATQGAGIVGKSASASQATPTVTIGSLTC

>Luke-3-7-N ACA1_031530

CSVTVKQTLGSAWTSNGQAYSQWNAQLVAGSEAVTSVTLSITGGDLEQLWELVKEADGSYRLPDYRIQNGGIVAGQVHQFGYIIKTSAQATITVTAKSC

>Luke-3-7-M

CTMKVTQTTRTTTDGHWTSGDFQFQIYDLVITNNGSYPITQGEISFQLAGDDDLIYQFWNLQREAADIDVFIIPTAYGPIQVGASQGAGYIVRSPLSAGQQTAPGFIPGVATC

>Luke-3-7-C

CAAKAALVARSEAQGGKWDDGAFSFQIYDLTINNAGSKPINGAQLTFTLASGTSISQWWELNQVSAAVFNVAFNYGNLQVGASQGAGVVERYAKGTTPDAPTVALGSVTC

>Luke-3-8-N ACA1_160160

CQVTVKQTLGSAWTVNGQDYSQWSVTLTNSGSEALKSVDLSIINASLFDQLWNIVKDNRGLYVLPDYILQNGGIAVGGSYQFGYIIKTKTQASIDIEKADC

>Luke-3-8-M

CAVTVESVARSAASGGQWTEGDSKFQIYDLTITASGNTPVDGVKLSINVAADVSIYQFWNLQRVDTSAIFTVPPWGSIQVGASQGAGFIVKSKIGASTAAPVVKVESTTC

>Luke-3-8-C

CAAKVSVVARSAASGGQWQDGANYNQIFDVTVTNIGTKAITGGQVQFALASGVSITQFWELNRKDATTFAVPTTYGPIQVGASQGAGIVGTSTSSATIAVPTATLTGLTCN

>Luke-3-9-N Neff_2021

CRVSIKQTLGSAWQQDGKNISQWNAQLTAGSEAVKSLQLSITGAKIIQLWELTNQSTGLFALPEYRIQNGGIVAGQTHQFGYIWESSSQATITVSSIDC

>Luke-3-9-M

CSLTVEQTTRSASQGGSWTAGDFQFQIYDLAFVNAGQQLVTGAQITIGTTDQQQIYQFWNIERKSADSASFNVPTSWGPIQLGASLGAGYIVRSPVSAGQLGAPSVTLDTTTC

>Luke-3-9-C

CSATVSLVARSAANGGQWVDNGATFQIFDITITNNGQRALNGGVATFTLAEAGATITQFWELNRQGSTNAFNVAFNYGALQAGATQGAGIVVRLAGSSAAAKPTVTLGSLGC

>Luke-3-10-N Neff_2021-tblastn

CSVSIKQTLGSAWQQDGKNISQWNAQLTAGSEAVKSLQLSIAGAKIIQLWELTNQSTGLFVLPEYRIQNGGIVAGQTHQFGYIWESSSQATITVSSIDC

>Luke-3-10-M

CSLTVEQTTRSASQGGSWTAGDFQFQIYDLAFVNAGQQLVTGAQITIGTTAQQQIYQFWNIERKSADSASFNVPTPWGPIQLGASLGAGYIVRSPVSAGQLAAPSVTLDTTTC

>Luke-3-10-C

CSATVSLVARSAANGGQWVDNGATFQIFDITITNNGQRALNGGVATFTLAEAGATITQFWELNRQGATNAFNVAFNYGALQAGATQGAGIVVRLAGSSAAANPAVTLGSLGC

>M12-1-N ACA1_379140(c)

CVKPTISQSRQTSWTDGLGVTYSVWSASIAAGSNTISSLILSIDPAANPAPIDNIWELVPKPGNPSLYELPQWRQQNGGIAAGQSHNFGYTIRSAQAAPFQVSSWNC

>M12-2-N ACA1_157520(c)

CKPLITQTKQTSWTDGSGVQYSVWDAFITAQSAPLTSVTLSLDAATGAQIDQVWEMVQPVAGVPLYTLPAWRLQNGGVPAGQTHRFGYTIRSAQPAPFDLSSWSC

>Dd-3-1-N DDB_G0292054 carbohydrate-binding domain-containing protein [Dictyostelium discoideum AX4]

CPLVNHCEKVNEWVENNDVPYAKFNCHITNIGNTPIGNVVMRLSGDGKTKEVWEMETKDNGQTYNFPQWRQQQQLAFNQSHPWGYTVQGGKELNVNLC

>Dd-3-1-M

CNLSNKCTVSNQWKNGDQVNTQYKCFITNNGKVPVSFIDIRINGNSDLYNVWEIITPNNGLSWDLTSWRIDKPLEATQSHEWGYIIVGDKPLDIQVC

>Dd-3-1-C

CTVSNKCTVEKEWKNGEQKNKQYSCEITNNGKGPLSHVDIRLSNNSALYNVWEVYTVNNGLSFDVSPWRLDNPIQPGQSHSWGYIVNDDVALDVKVC

>Dp-3-1-N DICPUDRAFT_91957 [Dictyostelium purpureum]

CPAKNKCERVKTWQESGQTLTKFNCYIKNIGRHPLGNVAINLDGAQVKEIWEVESSDKGKTFNFVEWRRSSPLGFGDVHSWGYIVYGKEPLEVNIC

>Dp-3-1-M

CKLTNECKKANSWVNGNITNTQFECEIKNIGNEPYSFIDIRINDNAALYNVWEIITENYGISWNLVNWRIENSLQPGKTHKWGYIVESDKPLDVQVC

>Dp-3-1-C

CFVNNKCTQDNQWKNGEQVNTQYSCKITNNGKVPLSHIDVRLNNNTELYNIWELQTSNYGISFDLPDWRIQNALEPGQSHSWGYIVTDNKELDVKVC

>Pv-3-1-N CYY_001342 [Polysphondylium violaceum]

CPVTNRCERVSSWIENGERLSRYSCFLKNVGPDEVANVAMTLSGAKIKEIWEIVTSDKG

KSWNLPEWRKTTPIQTGDENSVHSWGYVAYGDQALKVEIC

>Pv-3-1-M

CNLSNKCTLTNQWKQDGKVHTQFNCEIKNIGPKAISHADIRLNDNQDLYNVWEIRTLNNGLSYDLTNWREMKPLESGDSHFWGYIVKAEEALNVVVC

>Pv-3-1-C

CNATFEQKVVNTFIKDYVKYSQVEVTIKNSGQSSLSSVIVFSDAKFNSSWGIKQVDPINAPGNYQVLRNPKGLDVGSDEKFGYIFISNDGKAAPMNLISNAC

>Tl-2-1-N DLAC_04975 [Tieghemostelium lacteum]

CPVKNDCQRVRRFEEHGYPYTEFECILTNYGYLDVVNVDMRLDGARLKEIKGMETSDKGFSWNFPAWRKQRPLATGDSHRWSYIVNGYEPLRVTLC

>Tl-2-1-C

CSLDNKCTMVKQWVTDNKPYYQYTCQVINNGVAPVTHADVRFVNATSLYAVWELQTSNNGLTYDFVDWREQRPLEHGQTHMWGYIANENLAIQVC

>Pv-2-1-N CYY_001342 [Polysphondylium violaceum]

CTLSNKCTQVHQWMNDGVMNTQFACEITNMGSKPVTNADIRLNDNQNLYNTWEIATS

NNGLSWDFVKWREQKPLAVGLSHSWGYIVKSDKPLNVEVC

>Pv-2-1-C

CESTLVQTIVNHYMGKDHLKYTQVDVTVHNTGEKSLTSVIFFSDVHFNSTWGMTEVDRINAPGNYEVIRKSSSLAVGSQQKFGYVFVSDNTAAPINLISNAC

>Pv-2-2-N CYY_009706 [Polysphondylium violaceum]

CTVSNKCTQVHQWMNDGVMNTQFACELTNMGSKPISHVDIRLNDNQNLYNTWEIATS

NNGLSWDFVKWREQKPLEVGLVHKWGYIVKSDKPLNIEVC

>Pv-2-2-C

CEATFVQKITNYFLGKDHLRYTQVEVTVSNIGKKDLSSVIIFTDVHFNSTWGIKEVDPIN

APGNYEVIKKSKSLAAGSDQIFSYVFVSDNKVAPMNLISNAC

>Cf-3-1-N DFA_00997 [Cavenderia fasciculata]

CFLTNYCDLNKSWYEGGRVLSEFKCKLTNTGPFAIDHASIYLTGDYIKEL

NGLQTNNNGLSWDLLPKASLASGDQYSWSYKIFGTHPLIIDTC

>Cf-3-1-M

CNLSNDCQKDKSWTDNDRVYTQYKCSITNMGNTPVSHANIKITNNQNLVQISELTTNDHGVSWDFMNWREQTPLAAGQKHGYNYIVLGTEPVTISVC

>Cf-3-1-C

CTAAYTQKKDNTWTDGGKKYSQFSVTIKNTGTATLFSAITYTEAPLINFWGAVKVENFNYEMGSTATTAGVLAGATHTWGYVVEGETEARLNIVSNAC

>Ha-5-1-N PPL_01413 [Heterostelium album PN500]

CGCTYTCDNGIIGADGLGNTITKFTCMIINNGGAVDHIDVTIQNAANLVSLDGLQTSDGVHFDFQPSRVGSPLQTGNAHPFEYIVSGTTPLRFNIDC

>Ha-5-1-M1

CALSNSCQVGSSYQQNGQTYTVYNCMILNNGPAVSHTSIQLLNNANLVAVDGLQTSDGINFDFSPSRDSSPLQTGHGHPWSYTVVGNQQITVQIC

>Ha-5-1-M2

CALSNSCQVGSSYQQNGQTYTVFNCMILNNGPAVSHTSVQLLNNANLVAVDGLQTSDGINFDFAPSRDASPLQTGNGHPWSYTVKGNQPITVQVC

>Ha-5-1-M3

CNLVNQCELGNTYQKDGETLSKFFCMILNNGPTITHTSVQFLNNLNLVAVDGLATSDGLNFDFFPSRENSPLQTGNGHPFTYTVKGTNPVTVQVC

>Ha-5-1-C

CDASYYQTNYNTWTGPDGKTYSQFDITLKNIGTQPLKSVIVYAALEPFNNYWGALKKEAHNYDISAEKAAQTIAVNDQYQWSYVVEGSAPSALNIVSVAC

>As-6-1-N SAMD00019534_036040 [Acytostelium subglobosum LB1]

CLVGNSCLLDTTYTVSGQTRWNCNVINLGTPVNHIKLVLQDNAGILSVDGLKLAADGIVFDFFPDRSMNALQTNGNHPWSYITRGDQPATVYVEC

>As-6-1-M1

CLVGNSCILDTTYVVAGQTRWSCNVINLGTPVNHVKLVLQNNAGVISVDGLKLAADGIVFDFFPDRSMNALQTNGNHPWSYITYGDQPATVYVEC

>As-6-1-M2

CPLSIQCQIDANFHTVGQTKYNCMVLNNGAAAISMANILLSNNANLVSIDGLTTSNG

INFDFFPSRAQSALQPSHGHPFTYVVNGVSPLMFNAC

>As-6-1-M3

CQFSLSCAADSDFSVAGQTRFNCMILNLGSPAARINVNLFNNANLLALNGLTTTDGANFDFFPSRATSALQTNHAHPFNYIVSGNQQIGINLC

>As-6-1-M4

CTLANVCYQDSQTTENNQLYTKFTCEITNLGNFAISHTNVNFDDNSNLVKID

GLTTIDAGLSFDFLPHVEQTPIEIGAKYSWSYVVKGSSPLRVNIC

>As-6-1-C

CDATFSQSLSNTFSQDGCTYSQFNVTIRNIGTQALKSAIVYTAAEPLDNFSGARKVEAHNYDISEEAAAKTVMVGNTYTWSYVIKGSAPTAINIVSVAC

>Pv-1-1-N CYY_003620 [Polysphondylium violaceum]

CSLTNKCVQAHQWMNDGVINTQFTCEIKNMGSGPIKNADIRLDNNQNLYNVWEIMT

PNNGLSYDFIKWREQKPLAVGEVHQWGYIVKSDKPLDVTVC

>Cf-1-1-N DFA_03926 [Cavenderia fasciculata]

CPLTNRCYYVKSWTEYDLPVTQYNCSLTNYGRDAIGSINLGIYGSYIKDIWELTTNNGGKSWDLVEWRKQTPIQPGQAHTWGYNVHGQQELYINPC

>Dd-C1A-1-C1 DDB_G0281077 [Dictyostelium discoideum AX4]

CSVNITQTIINSWNDGNQDFTQVQVIITNNGPTTIRSFSFKLNNIISIWEVETLSSNSYQLPNWVGNISSGSSHSFGYIQQSNQTTPLTDETSTC

>Dd-C1A-1-C2

CNANVTQTITSSWRENNQDYIQVETIITNIGQSIIQSYTFELNDIQSIWEIQTLSNNTFTLPNWKNSIEVGSSHTFGYIQKAYSINPLLSVNKVC

>Dd-germ-1-C1 DDB_G0291856 germination protein [Dictyostelium discoideum AX4]

CNVSVTLKVTSQWVENNQNFIQVEATIFNYGQSEITGFSFQLFNIISIWNVDQTSFNSYSLPIWLPAIQVGSSFSFGYIQKTDTVVPLSSQSVVC

>Dd-germ-1-C2

CMINVSQSIVNSWRENTQDFIQVEVNLKNIGQNTVYQFSFEIFDLVNIWGVELLSNNTYQLPSWVYSIPVGSSHNFGYIQKSNHLLPIYSQLPTC

>Dd-ND-1-C DDB_G0282255 carbohydrate-binding domain-containing protein [Dictyostelium discoideum AX4]

CSANVTQKIINQWYSNNVYYIQVDATINNKGSKPVRGFNFQLQNVQDIWNADQTSTNNYKLPSWLGEIPVNGAHQFGYIQRSTSLTPITGTPSC

>Cf-2-1-C1 XP_004362743.1 cellulase [Cavenderia fasciculata]

CAVKTTQTLSNSWKADGVNHYQYVVSVLNQADLPLTDTLHIIIANPDKLLNTWEITKMQDGVYTLPSWRGVSPVLKGESHNFGYITTSPTANTISIDGC

>Cf-2-1-C2

CHATLSQAIVNSWTNDGKNYVQVQATVENTGTKDLKNFKATLPSDQLSQIWQMDKDGTDQYQLPSYQDKIAP

GQSYGFGYIALSSTNTALEASNVVYSC

>Pf-GH16-1-C1 PRP88067.1 glucan endo-1,3-beta-D-glucosidase [Planoprotostelium fungivorum]

CIDVQQTKTASWREGNRQVFQYEVTVKNSASVSVEILLMAPSAQSDKIRQIWNLEQNGTKSSQSHWLLPQWQHGINPHSSFTFGYISLDSSVSFVSSPFTQC

>Pf-GH16-1-C2

CGLYTNQTLISSWYQGSTKIVQYEVKLTNEGTVSRQPYFTTVDESKILSIWGLTDVGVNSWSLPDYNKKIDGKSTYTWGYIASADQILFSVNKSKSNC

>Dd-1-1-C DDB_G0269112 cellulose-binding Protein [Dictyostelium discoideum AX4]

CLAQVQQKVINSWINGEVDHYIQVEATIVNQGSTPISSFNFYSDAEQIWSVEKTGTNTYKLPSWFSTIPVGGSHTFGYIVKSAELSDLEGVQYTC

>Ha-GH9-1-C XP_020433331.1 cellulase 270-6 [Heterostelium album PN500]

CQGKISQNVVSTWLNNGVQYKLVNVEITNIGTSKINSLQFNLDPKIQQIWGVTNTGDVTYTLPDYQVGLRVGETFVFGYIIQTTINVEYSNDQYTC

>As-GH9-1-C

CSVAFHQRVLNQFQSGSETFSVVDTIVTNDGSAKVKSLVFEISQPVESIWGVSTVGKKYSLPSYLKEVNVGETFSFGLIVKGTVPPGIINKVLKC

4DKs of Leo-A and Leo-S

>Leo-A-1-N ACA1_074730

CPGSDFFPDQRGCCPVVVNSVPFYADAQGCFPRIISGVAFYADAGGCYPSSAGVYRSAGAVCPADRVCEPEC

>Leo-A-1-C

CANGFFSDVNGCCPRVLVDNVPSYRDAQGCYPISIASVIFYADVGRCYPNSSGVYRPAGAVCPAGQQCVVSC

DRNYA

>Leo-A-2-N ACA1_083920

CPGSDFFPDQRGCCPLVVGSTPFYADAQGCFPRFIGGIAFYADAQGCFPNSAGVYRSAGEVCPADRVCVPQC

>Leo-A-2-C

CANGFFSDVNGCCPRVLVDNQPSYRDAQGCYPISIASIIFYADVGRCYPNSSGVYRAAGEVCPANQVCVVSC

>Leo-A-3-N ACA1_394030

CLGGAFPDAGNCCPTMINMINFYRDARGCYPTRVANNQVLYADVGGCFPNAQGTYRPAGATCPPGQQCSPTC

>Leo-A-3-C

CPANAQRDPAGCCPRAFGMSLFYRDVRGCYPIATNVGVLYADASGCYPNEAGVYNPAGMACPAGRQCVPACD

>Leo-A-4-N ACA1_093360(c)

CPMGFWPDGRGCCPEVINGLPYYRDGRGCYPRVFDGVAYWPDGNGCYPDSQGHYTKSVVCPVAQKCWSSC

>Leo-A-4-C

CGGSGLGPDENGCCPRVVAGVPYYRDRRGCYPILIAAGVRYADGLGCYPDAKGRYRDNAFPPVVGFDGPSLGCPEAQVCRPAC

>Leo-S-1-N ACA1_188350

CPGGFWPDGSGCCPLIIKGSPFYRDQNNQCYPRQIGSSIFYADSNGCYPDANGRYVSAGESCPSDQKCQSMC

>Leo-S-1-C

CPGDKKKDRNGCCPRLINGKWYYRDGRGCYPTQTPKGVFYADGNGCYPNDHGVYLKAGQSCPHERKCMAECDC

>Leo-S-2-N ACA1_188550

CPGGFWPDASGCCPQLINGFGYYRDVKNQCYPKSIGGHVFYADSNGCYPNEQGVYNPAGQSCPSGQQCHPSC

>Leo-S-2-C

CPGGQKPDRNGCCPRLINGKWYYRDGRGCYPTQTSKGVFYADGNGCYPNDHGVYLKAGQSCPHERQCMPEC

>Leo-S-3-N ACA1_374130

CKGGYYPDRKGCCPAIIKGKAYYRDGNKCYPKDVGCGVYYADGHGYYPDEKGNYAKDGEECPKERKCHERC

>Leo-S-3-C

CPGGQWKDGKGCCPRKIGHQWYYRDAHGCYPVERDCGVFYADGKGYYPDENGQYAKAGEYCPEYRKCKPEC

>Leo-S-4-N ACA1_291580

CKGGYWPDGKGCCPKIIKGKAYYRDANKCYPKDLDCGVFYADGNGYYPDEKGNYAKDGEHCDDERKCHQYC

>Leo-S-4-C

CHGGQWSDDDGCCPRKIGHQWYYRDAHGCYPVERDCGVFYADGQGCYPDEHGKYREYGEKCPRHRQCKREC

>Leo-S-5-N ACA1_116240(c)

CKGGYWPDGKGCCPEIIKGRAYYRDGNKCYPKDLGCGVYYADGRGYYPDEKGNYAKDGESCSWERKCHERC

>Leo-S-5-C

CPGGQWKDGHGCCPRKVGREWRYRDAQGCYPIELDCGVFYADAQGCYPDEHGKYLKAGECCPDHRKCCPEC

>Leo-S-6-N ACA1_188370(c)

CKGGYWPDGKGCCPEIFKGKAYYRDYNKCYPRDLDCGVFYSDEDGCYPDEKGEYLEAGEWCPKDRKCHERC

>Leo-S-6-C

CANGQWKDGKGCCPRKVGDSKYFYYRDGRGCYPIDTKRGVLYADSNKCYPDNNGKYLKAGEYCPEDRKCKPEC

>Leo-S-7-N ACA1_365840

CKGGYWPDGKGCCPKVIKGKAYYRDHNKCYPRDLDCGVFYADEERCYPDEKGQYLKNDEWCPKDRKCHERC

>Leo-S-7-C

CPGGQWSDDDGCCPRKIGHQWYYRDAHGCYPVERDCGGVYYADGKGYYPDEHGKYAQAGEYCPDNRKCKPEC

>Leo-S-8-N ACA1_117050

CKGGYWPDGKGCCPELINGKAYYRDGNKCYPKDVGCGVYYADGNGFYPDEKGNYLKAGESCSWERKCHERC

>Leo-S-8-C

CPGGQWKDGHGCCPRKVGHEWRYRDAQGCYPIELDCGVFYADAQGCYPDEHGKYLKAGECCPDHRKCCPEC

>Leo-S-9-N Neff_2021-tblastn

CKGGYWPDRKGCCPEIIKGKAYYRDHNKCYPRDLDCGVFYADEEGCYPDEKGQYLKDDEWCPKDRKCHERC

>Leo-S-9-C

CHGGQWSDDDGCCPRKIGHQWYYRDAHGCYPVERECGGVYYADAKGCYPDENGKYRAAGEKCPHHQQCKREC

>Leo-S-10-N ACA1_096640

CKGGYWPDGKGCCPKIIHGKAYYRDGNKCYPKDVGCGVYYADGNGYYPDEKGNYPKDGEDCDDERKCHKYC

>Leo-S-10-C

CHGGQWSDDDGCCPRKIGHQWYYRDAHGCYPVERECGGVYYADGKGCYPDENGKYREYGEKCPRHRQCKREC

>Leo-S-11-N ACA1_390440

CGADFFSNGDGCCPIFLNGSAYYPGADGCYPILTDLGVQYSDEFGCYPDLEGFYEGVENCTNPCAPEC

>Leo-S-11-C

CESTNLYRDANGCCPALINGVAVYRNAEGCYPINTSVGIVYPDANGCYPNDDGVYVAEGEDCPEAQQCAAQC

BHFs of Jonah lectins and selected bacteria

>Jonah-1-1 ACA1_164810

CPAEDFFGPAADLNVLVTGSDKLIYDLDMVNGDVEGRVAVNGGFRVKSFGTAQAYSCPDTANYASMFNLIVNGKMDYSNGQLFCGSSISLSSMNDPSLRQKDFGATNARVAAGKTIKDVTGFDFVATAGYLQGVSNYFSKYAPGTHSHFRVTEVSPADVNKLNIEGGDYVQVINIKGSGDINFSNFEIPTALNPSQVIYNVVGNNNIKISGFGLKGHLLALNSDVVLENGHVAGNVYVRSLVGQGSGQVNLAPCP

>Jonah-1-2 Neff_2021-tblastn

CPQPLLGVAAGFSAFVFGNETDGSSNATTFSVDLVNGDFESAVAVNGGLRLRSFGIFQNSPCTDQASQGSTLIVNGPVNAVDTHLFCGNLLVANQSDVIRPPSFQPGNGVVASNVSIDFSSVRTNHTGVSAVLCALTNATPADTTIFGDVRLRATGLETEVFTVDVSTLARTGFMRLTNVDASVTKSVIINVLGSGTARFSNFFFDTAAIPQGLITWNICSADRVVIENFLFPGTLLAPAASVTLQKAQVVGSVVASSLTGSGGGQINVARCP

>Jonah-1-3 ACA1_261530(c)

CPPNTRLAGLIDLYSAFILGNQSSNATSLDMMNGDFENRLAVRGGLRLRSFGVNQRAVCTAEESAGFGIVAQGPGPIDFANGRLFCGNLLASNDSQIVNLPSFANNAGLVVGNVTERSGINFTLDAAELRNVSSNFCNASQTNATSAEVAEFGAITLRATGAANESFNVQASDLARSTGMRLVGFGSSTEEVVINIVGNQTATFSNFFVDVGSVPLTSIIWNVCEANLVVIQGFQFPGILLAPNSDVTLQNGLVLGQIVAATLRGSGGGQINVPFCP

>Jonah-1-4 ACA1_133400(c)

CPPNNARLGQAADFSAFVLGNSSAVGPSLDMVNGDFESAAAVRGGLRLRSFGVNQAVSGSACTDGESQRFTLIAEGGTIDYSNGQLFCGNLLASADAEILSAPSFGGGSVKVGDVTELSGIDFEEAGRELLDESETLCSAVEDGEATDAEVDSSGGITLTATGATVEVRLLPHQRINQRTNAQCFANSWRFFNKQMFSVRARDLSAATGVRFVGFEGQSINEVIINVMREADDSEASLVLSNFAVDLGSVPVRSVLWNICGGTDLVVIQAVGLPGALLAAGADVTLQNGQVTGQVVARTMRGSGGGQIINPAC

>Jonah-1-5 Neff_2021-tblastn

CQPALLGGGFSAFVLGSASASPNAAALDVANGATGSALAVRGGLRLSSFDINNGHSQVCDDEDFGVLVEGSGVVVDVTNSQLSCGGLLASTNANIVSLPSFVGSAGLTVGNVQALSGISFALAEAKLSTTSQRLCNATPSGTVQVTPWGAITLTGAINQLNQTFRVQTSNLATATSMQLVGIGALKLQSVVINVVVASAEEIVSLSNFFVDLGSVPVQTIVWNICGAPDVVISGFQFPGVLLAPRSNVTLENSQVHGQVIAKTLQGGSFQITPSCT

>Jonah-3-1-N ACA1_121780

CPPSLLGSASAYNVFLLGSSAANGYTFDLINGDVEGRVASNGNFRAVNFGVGTRLGNCNASTPTVIIGGASSSVSFSGGLQCGSLVTESSATIVTLPGFANNGQLVQGSVSDLTGIDFAAAKTELQAQASSLCSAGGIGATRSGNAITLQGTSGASQAFTVNVADINVASSLTLTVPDASAVEQVVVNIVGSGAINMGGFQTFLNGVSSGLVTYVVCEAPSVTIKNFGLYGNLLAPSSTVSIQNAQLLGNVVALTLTGTGSGEVHVPTCP

>Jonah-3-1-M

CPAPLFGSGVSVYNVFLLGREGANYTFDMTNGDVEGRVATNGSLRAINLGVGSRIVPCNASVASVIVAGEDSSINFVGGVQCGTLVAENTADIVTLPGFANGRPVLGSVQNITGVNFTAEAEQLNATANALCAAGGVPATVSPWREISFAGSANASQAFTVNASDINVASSATFNFPNASAVQSVVVTIVGSENLTFTNFGINNGGVPGAYITYVVCSAEQVTVRGFGLEGNLLAFNSSLIVENGQIVGNVVAQTMSGSGGGEVHVPECP

>Jonah-3-1-C

CPPSFFGGAAAYNVFLSGNADKEFAFNMINGDVWGKVAVDGSVFVRSFGFGTRLTCSGDAANQATLIASGADVDVTGEIQCGNLVTANTTTVTGVSFRNGQAIAGDITALTGLDLAAVASDLASGSSSLCSVEGTAATVGAGNRITFAGSDSASQTFTITTAQLAAASWISFAFDDASAVQQVVVNVIGDDDVEFSGFDLNIGGVANGRMTWNVCSATSVSVSSFNFQGNLLAPLADVSLTNAQINGNVAARTLTGTGSGEVHLPQCP

>Jonah-3-2-N ACA1_261090(c)

CPASFFGSAASGYNVFLLGGGSGNYTFDLINGDTEGSVAVNGNFRATNFGVGTRLGSCNASTPTLVVGGEGSSVSFSGGLQCGSLFAEESAQLISLPGFANGELVQGSVLDNTGIDFGAAQTELEDVASSLCSAGGVAAGVSGGAITLAGNGNASQAFTINVADINAASSLTLNVADASAVDQITILLVGSGAINMGGFQTFLNGVSSGQVTFVVCAAPTVNIQNFGLYGNLLAPFSAVSIQNGQLLGNVVAQSLTGTGSGEVHVPVCP

>Jonah-3-2-M

CPAPLFGSGVSAYNVFLLGAPGASASNFTFDMTNGDVEGRVATAFGSLRASSFGFATRLTGCNSSDATIIVGGANATINIFNAGVQCGSLVAENSADLISLPGFANGRPLLGSVQNLTTVNFTAEAAQLNATANQLCAAAVNSTAATVSPWREISFTSTSANASQAFVVNASDINVASSATFSFANASAVESVVVAIVGSANESLTFTNFGINNGGVPGAYITYVVCSAPSVTVSGFGLEGNLLAYNSSLTVENGQIVGNVVAQTLSGSGGGEVHIPVCP

>Jonah-3-2-C

CPPSFFGGAAAYNVYLTGNPSRAVAFNMTNGDVWGKVAVDGSVVARNFGFGTRIQCTADAANAATLIASGSSVSFTAGAVDCGNLVTPASTTVSGVNFNNGKAVTGDIETLTRLDFADVSASLSSASSALCSAEGIAASVGSGNRITLASTASGAAQITFTLTTAQLASASWVRFAIADVASVKQIVVNLVGDDAVDFSGFDTDLGGVSNGLITWNVCSATSVTVTAFNFQGNLLAPTADVSLSNAQINGNVAARTLTGSGSGEVHTPVCP

>Jonah-3-3-N Neff_2015-tblastn

CPPSFFGSASAYNVFLLGGGSAANTFDLTNGDVEGRVASNGNFRAVNAGVGTRLGNCNASTPTAVIGGASSSVSFSGGLQCGSLVTESSATIVTLPGFANNGQLVQGSVSDLTGIDFAAAKTELQAQASSLCSAGGIGATRSGNAITLQGTSGASQAFTVNVADINAASSLTLTVPDASAVEQVVVNIVGSSAINMGGFQTFLNGVSSGLVTYVVCEAPTASFTNFGLYGNLLAPSSAVTIQNAQLLGNVVALSLTGAGSGEVHVPTCP

>Jonah-3-3-M

CPAPLFGANVSAYNVFLLGREGANYTFDMTSGDVEGRLATNGSLRAISFGVGSRIVPCNASVASVIVAGENTTINFQGGVQCGTLVAENSAELISLPGFANGRPLLGSVQNITGVNFTAEAEQLNATANALCAAGGVPATVSPWREISFAGSANATQAFTVNASDINVASSATFNFPNASAVQSVVVTIVGSENLTFTNFGINNGGVPGAYITYVVCSADLVTVRGFGLEGNLLAFNSSLIVENGQIVGNVVAQTMSGSGGGEVHVPECP

>Jonah-3-3-C

CPPSFFGGAAAYNVFLSGNADRQFAFNMTNGDVWGKVAVDGSVFVRSFGFGTRLTCSGDAANQATLIASGSTVDVTGEIQCGNLVTPASTTVTGVSFRNGQAITGDITSITGVNFGAIESALSSASSSLCSAQGTAPTVGAGNRLTFAGSDSASQTFTITTAQLAAASWISFAFDDASAVEQVVINVVGSDAVEFSGFDLDIGGVANGRMTWNVCSATSVSVSSFNFQGNLLAPLADVSLTNAQINGNVAARTLTGTGSGEVHLPQCP

>Jonah-3-4-N ACA1_157320

CTTALFNGAQSFDTFLFGSATIQNGQFGGRVAISGNANLTQFGIANALPCTNADARNFNLIVNGNLTSVNGQVACGSALVTGTVVSAPNFRQEAQGAGVVSGSASMAAVDFDQSFSSLLFTNQQICSLTCKQAAITGSGAVTFDFDNTTNVYCLNAADASRATSFRFTNFPVDFAGALAINILGSAGETVTLSNAATNLGNVKPTQIVWNVCNGLNVRLSAFTLFGSLLAPASSINITNAQQTGITVGRSLTANGFQKFQSLFSGFFCL

>Jonah-3-4-M

CSSPIKALDGAADFDTFVLGQGDTLTARNGQFGGRVAVNGNARLSSFGMGAELVCAANDSSAFNLIVQGNLSATNGEIFCGSALVNGSIQSAPSLRQSNQGANVTQGDLLTLSTLDFTESADNLTRINRQLCEAFADNCTQAEIDAFGIVSLRWDNLTNGGRAQVFCLNSSLIANATFFEFAGFPANFSGAIVVNILGSEDVRFANAAISAGNLNATQVLWNVCNATRVDIFAFQLFGSLLAPLSNVSLINAQQIGTTVALNFTGSSFQKLLAPFIGEIC

>Jonah-3-4-C

CTNALFGAAQAFDLFVLGTGDQNVTTGLNVVNGQFAGRVAVNSGARLNDFGIGNSLNCTAANATNFNFIVQNDLNATNGELFCGSALVGGNVTNPLTFRQVNEGANLVSGVDVSISGIDFASATSFLTGVNQAVCALNCTNVTLGQANELRFTGAAGANSTQIFCVRSADLSRAASIVFDFAANFTAPIVVNIQGSPNDTTASFRSAAIQRGRLRSEQLLWNVCDSLDVTLSQFQLFGSLLAPNSSLTISNAEQVRTQASHFGPHARTHAPDRV

>Cyanobacteria-1 HBB36285.1 [Cyanobacteria bacterium UBA9273]

APLSLAATMALGFTTQASAISLGPAADYNVFVLGDVYQTNTDIEGKLAVGGNVLLSSFGVGDKIQSGDVLIAGGNLDLTN

GQIYGNAIYGGSKVISKTDLNSKGANVTGTLLQGNPLNFEEAGNYLRDLSASLSQQTVNGTTTINNNAIALKGTDSKLNV

FNLSGAQLKTANSFSIDAAKDSTVVVNISGADIDIGSFGFFLNNGLSSQNVLYNFYEATNLKSSGSGFEGSFLAANADFT

FNNGQINGNLIVKSLTGTGESHNNL

>Cyanobacteria-2 HBE20857.1 [Cyanobacteria bacterium UBA11367]

LGVSAPSHAISLGPASDFNVFVLGDIEQNFVDTEGRMAAAGNVTLTDYAVGSGFASNPTGNNLIVGGNLNFTRGTVFGNA

VYGGTATLDLVDVKGTSSQGNLIDFAAAGQELRNLSAYLGGLSANGTKTFEYGQVTLNGSGSGLNVFNLLGSELSAANTF

TINANPNATVLVNITGETISFGNFGFFLNGADKQKVIYNFVDATNLSSTGVGIKGSLLAPKAHYQFDNGHIDGNLIAGSL

KGGG

>Hyalangium WP_224363079.1 choice-of-anchor A family protein [Hyalangium versicolor]

VEVNLSDYNLFLLGDYNGGHDVVGKVAAGGNIVLSDFAVGSGLAASDTSNVLVAGGNLSVNRGAVYGDARYGGSYSTNPS

VVFPRGSAAQGTPIDFAARGAELSNLSAQLAGLNANGTKTRYNWGGIYLTGTDATINVFNVPASDFNGAALLDINAPASS

LVVVNITGASASFGGFGTQFSGGANQHNVVYNFVNATSITANGFGFWGTILAPNADVTFNNGSWDGGLYAKSLTGNAEGH

IN

>Bryobacter WP_031500423.1 choice-of-anchor A family protein [Bryobacter aggregatus]

DFNLFVFGNLTQANTDVQGRVAVGGTASVTNFGIGTSLSPDPSRLDLEVGGSLSWTNGQLFNGSGIYGGSAALTSVGVPN

GTITNVVPNINFAQVKTEETNLSAYLAGLSAITATATPWGALTLTGTNASLNVFNVSAGVLSGINTFTFNVPTTSTVIVN

IAGATNSLQNAGMNLNGLSKTQVIYNFYEATALTVQGISVQGSILAPNATLNFNNGNIEGQVIVNNLLGGG

>Bacteroidota MBU1678804.1 choice-of-anchor A family protein [Bacteroidota bacterium]

LGAAEGLNLFLFGDLSQPSADTEGRMAIGGNAHLSNYSVGAALSGSGNQDVLIVGGHLEFISGGVYGGNVVYGVSTNLHA

TYYPVTIFDGTLRQDTPIDFDAAEASLKSLSTDLAGYSATGSTDFEWGNLTLTGNDPFFNVFDLSGDDLSNAYGMTIDVP

NGSVVLVNISGDNIDWNGGLFVNGTALSNVLYNVYEANNLKIQGIDIRGSLLAPYANLHFPSGVINGQVIAQSMAGSGQY

NSGQFN

>Myxococcaceae HEX8705299.1 choice-of-anchor A family protein [Myxococcaceae bacterium]

VAGKVAAGGNITLDSFGVGSALAGTNITNTLVAGGNLTLSSGGVAGDTWYGGSYTPNWSVRISPGTLARGTPIDFEARFD

ELRSLSARLATRPINGATEPRWGNLYMTGTDPCLNVFEVRASDFKNAQGRNISAPAGSFVVVNIRGTALSFNGGVSFSGG

ITPQRVLYNFVEATNLDARGFGLQGTVLAPYAHVTLNDGSWSGGIYAVSLHGNATGNLSP

>Stigmatella SEM17179.1 choice-of-anchor A family protein [Stigmatella aurantiaca]

DVEGKVAAGGNIAMTHFAVGFKLSENQIAQTLVAGGDLTLSNGGIWGDAWYGGSYNADSGVLHPRGTLAQGSPIDFAARG

GKLRALSSALGSLTANGTVQQPWGVITLSGSDPQVNVFQMSAGSFTGATQLSIQAPAGSLAVINILGSSATFTGFSTELR

GGIDKRRVLYNFVDATAITAEGFGFQGTVLAPFADIQFSNGSFDGGIYARSLTGNAEGH

>Caldilineaceae MBX3001654 choice-of-anchor A family protein [Caldilineaceae bacterium]

AGDFNLFVLGDLSRSYTEAQGRVAAAGNTSLLGFDIGSALANSRGSRDDLIVGGNLTFSSGNLHNGNIVHNGSANINASV

GIPNGSARQGAAINFTKARQDLEGLATKLSGLPVNGTTTFQSGTLTLTGNNQALNVFTVDGRTQLSTTTSLRINMPAQAT

ALVNISGAAVNFQNATIFINNVSGDAPPGQQRILYNFYQATSLTVSGIGVKGSILAPYAQVNFNNGHLNGNFVAKSVANG

YGAVNQSP

>Nostoc WP_242072139.1 choice-of-anchor A family protein [Nostoc sp.]

AQDYNVFVFGDMNQSSDSEGRVAVGGNATFTNFGIADRLSNSNGTDTRLVVGGDLTYNGGQIFGGNAVVGGTVKTSVNFN

CSPNCGVNSGKPINFDAAKQELTYLSESLAGLSSTGTTEYKWNGIYLQGNNSDLNIFTIDGSQFSKSSYLNLSDVGTNST

IVFNILGNSVDISNFGLNLNNVNKSNILFNFVDATQVKTTGFSFLGSVLATKANVQFNNGNVEGTLVAASLSGSG

>Nodularia TVP54975.1 choice-of-anchor A family protein [Nodularia sp.]

AQDYNVFVFGDMNQSSDSEGRVAVGGNATFTNFGIADRLSNSNGTDTRLVVGGDLNYNGGQIFGGNAVVGGNVNTPVYFN

CSPNCGVTSGNPIDFDAARQELNHLSEYLGGLAATTTTEYKWGGIQLQGKKDDDLNVFTIDGSKFSSSTYLDLKEVGGNS

TVLLNILGDSVQIENFGFNMNGVNQQNVLFNFVNATQVTTTGFSFNGSVLATKAHVNFNNGNVEGTMVASSLSGSG

>Anabaena WP_015214753.1 choice-of-anchor A family protein [Anabaena sp.]

AQNYNVFVFGDMNQSSDSEGRVAVGGNATLTNFGIADRLSNSNGTDTRLVVGGNLTYNNGQVFGGNAVVGGTVNTPVYFN

CSPNCGVSSGKPIDFNAARSELTNLSNYLGGLVSTNTTEYKWGGIYLQGSNSDLNVFTIDGSKFSSSSYFNLQGVGSNST

VVLNILGNSVDIKGFGDLTGVNKENVLFNFVDATQVTTTGFSFQGSVLATKANVNFSNGNVEGTLVASSLSGSG
